# Pan‐Continental Genomic Analysis of Eurasian Perch Uncovers Global Diversity Hotspots and Postglacial Recolonization Patterns

**DOI:** 10.1002/ece3.73502

**Published:** 2026-04-21

**Authors:** Vitalii Lichman, Mikhail Ozerov, María‐Eugenia López, Kristina Noreikiene, Siim Kahar, Lilian Pukk, Oksana Burimski, Bella Japoshvili, Dijana Blazhekovikj‐Dimovska, Dmitry Lajus, Dušan Nikolić, Filipe Ribeiro, Tatyana Gebauer, Jan Kouřil, Jiří Peterka, Petr Blabolil, Martin Čech, Tomáš Jůza, Jan Kubečka, Milan Muška, Marek Šmejkal, Mojmír Vašek, Kimmo Kahilainen, Linas Ložys, Jens Carlsson, William Corcoran, Özgen Yilmaz, Peter Eklöv, Péter Takács, Bálint Bánó, Réka Pallos, Stefan Kazakov, Luchezar Pehlivanov, Thomas Lecocq, Sophie Lambert, Torben Lauridsen, Andreas Berthelsen, Pedro Raposeiro, Hugo Verreycken, Robert Britton, Jost Borcheling, Yuliia Kutsokon, Oleksandr Didenko, Pavel Jurajda, Rafael Miranda, Riho Gross, Anti Vasemägi

**Affiliations:** ^1^ Institute of Veterinary Medicine and Animal Sciences, Chair of Aquaculture Estonian University of Life Sciences Tartu Estonia; ^2^ Biodiversity Unit University of Turku Turku Finland; ^3^ Department of Aquatic Resources, Institute of Freshwater Research Swedish University of Agricultural Sciences Drottningholm Sweden; ^4^ Department of Biology University of Turku Turku Finland; ^5^ Institute of Biosciences, Department of Botany and Genetics Vilnius University Vilnius Lithuania; ^6^ Institute of Zoology Ilia State University Tbilisi Georgia; ^7^ University “St. Kliment Ohridski”, Faculty of Biotechnical Sciences Bitola North Macedonia; ^8^ Estonian Marine Institute University of Tartu Tallinn Estonia; ^9^ Department of Biodiversity and Environmental Research University of Belgrade – Institute for Multidisciplinary Research, National Institute of the Republic of Serbia Belgrade Serbia; ^10^ University of Lisbon Marine and Environmental Sciences Centre (MARE), Faculdade de Ciências da Universidade de Lisboa, Campo Grande Lisboa Portugal; ^11^ University of South Bohemia in Ceske Budejovice, Faculty of Fisheries and Protection of Waters, South Bohemian Research Center of Aquaculture and Biodiversity of Hydrocenoses, Institute of Aquaculture and Protection of Waters České Budějovice Czech Republic; ^12^ Biology Centre, Czech Academy of Sciences Institute of Hydrobiology České Budějovice Czech Republic; ^13^ Faculty of Science University of South Bohemia in České Budějovice České Budějovice Czech Republic; ^14^ Lammi Biological Station University of Helsinki Lammi Finland; ^15^ Nature Research Centre State Scientific Research Institute Vilnius Lithuania; ^16^ UCD School of Biology and Environmental Science University College Dublin Dublin Ireland; ^17^ Inland Fisheries Ireland Dublin Ireland; ^18^ Alaca Avni Çelik Vocational School, Department of Veterinary Hitit University Çorum Türkiye; ^19^ Department of Ecology and Genetics, Limnology Uppsala University Uppsala Sweden; ^20^ HUN‐REN Balaton Limnological Research Institute Tihany Hungary; ^21^ Doctoral School of Veterinary Science University of Veterinary Medicine Budapest Hungary; ^22^ Institute of Biodiversity and Ecosystem Research at The Bulgarian Academy of Sciences Sofia Bulgaria; ^23^ Université de Lorraine, INRAE, L2A Nancy France; ^24^ Department of Ecoscience–Lake Ecology Aarhus University Aarhus Denmark; ^25^ CIBIO, Research Centre in Biodiversity and Genetic Resources, InBio Associate Laboratory, BIOPOLIS Program in Genomics, Biodiversity and Land Planning; UNESCO Chair – Land Within Sea: Biodiversity and Sustainability in Atlantic Islands, University of The Azores Ponta Delgada Portugal; ^26^ Research Institute for Nature and Forest (INBO) Brussels Belgium; ^27^ Department of Life and Environmental Sciences Bournemouth University Poole UK; ^28^ Ecological Research Station Rees, Institute of Zoology of the University of Cologne Rees Germany; ^29^ I.I. Schmalhausen Institute of Zoology, National Academy of Sciences of Ukraine Kyiv Ukraine; ^30^ Institute of Fisheries, National Academy of Agrarian Sciences of Ukraine Kyiv Ukraine; ^31^ Czech Academy of Sciences Institute of Vertebrate Biology Brno Czech Republic; ^32^ Universidad de Navarra, Instituto de Biodiversidad y Medioambiente (BIOMA) Pamplona Navarra Spain

**Keywords:** biogeography, genomic diversity, glacial refugia, phylogeography, population genetics, SNP

## Abstract

The contemporary distribution of genetic diversity in widespread freshwater species reflects a complex interplay between historical processes and recent demographic events. We investigated the postglacial recolonization history of the Eurasian perch (*Perca fluviatilis* L.) across its native range spanning Europe and Western Siberia, aiming to understand how historical and recent demographic processes have shaped contemporary genetic diversity in a widespread freshwater species. Using an integrative genomic approach, we combined whole mitochondrial genome resequencing with nuclear SNP‐array genotyping (3660 SNPs) for 382 individuals from 188 locations to reconstruct patterns of lineage divergence, population structure, and admixture. We identified five highly divergent mitochondrial lineages, consistent with the existence of multiple glacial refugia across Southwestern, Southeastern, and Central Europe, as well as Siberia. Nuclear data (3660 SNPs) revealed three major genetic clusters corresponding to Western, Northern, and Southeastern Europe, along with strong regional admixture. The Baltic Sea region emerged as a contemporary hotspot of genetic diversity, likely resulting from the admixture and convergence of distinct maternal lineages during the postglacial recolonization of Northern Europe. Signals of isolation by distance were evident both within and across lineages, highlighting the role of limited dispersal in shaping current genetic patterns. The integration of mitochondrial and nuclear genomic data provided a comprehensive view of the evolutionary history of *P. fluviatilis*, revealing both deep historical divergence and recent admixture events. The existence of multiple glacial refugia and subsequent secondary contact underscores the complexity of postglacial recolonization processes in freshwater fauna. These findings advance our understanding of how historical and contemporary factors interact to shape biodiversity across Europe.

## Graphical abstract

We reconstructed the postglacial recolonization history of Eurasian perch using mitochondrial and nuclear genomic data spanning Europe and Western Siberia. The analyses revealed multiple glacial refugia and extensive secondary admixture, reflecting a dynamic history of range expansion and contact among divergent lineages. Together, these patterns illustrate how historical and contemporary processes have shaped the species' complex genomic structure across its native range.

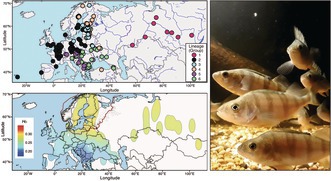

## 1 Introduction

The rapid progress in high‐throughput sequencing technologies has transformed population genetics and phylogenomics, enabling researchers to investigate genetic diversity, demographic history, and adaptive evolution with unprecedented resolution (Ellegren 2014; Goodwin et al. 2016). Genomic tools have been especially impactful in biodiversity studies, allowing detailed reconstructions of phylogeographic patterns across broad geographic ranges and the detection of fine‐scale population structure and signatures of selection (Ekblom and Galindo 2011; Ouborg et al. 2010; Edwards et al. 2022).

Despite these technological developments, many genome‐wide studies remain constrained by limited geographic coverage and small sample sizes, hindering our understanding of global genetic diversity patterns (Fuentes‐Pardo and Ruzzante 2017) and the reconstruction of postglacial recolonization routes (Emerson et al. 2010), particularly in species with complex demographic histories. Broadly distributed taxa require extensive and spatially representative sampling, but trade‐offs between the number of individuals, sequencing depth, and geographic scope can reduce the power to detect evolutionary processes across spatial and temporal scales (Ellegren and Galtier 2016; Bravo et al. 2019). Cost‐effective strategies, such as low‐density SNP genotyping assays (Andersson et al. 2024) and genome skimming (Chang et al. 2024), offer large‐scale assessments while maintaining population‐level resolution. Still, carefully balancing sampling design and sequencing depth remains essential for producing robust phylogenomic inferences (Fumagalli 2013; Shafer et al. 2015).

To effectively interpret genome‐wide data in an evolutionary context, historical processes such as glacial cycles and postglacial recolonization must be considered (G. M. Hewitt 2004b). The classical postglacial colonization model (G. M. Hewitt 1996, 1999; G. Hewitt 2000) proposes that repeated fluctuations in global temperature throughout the Pleistocene (approximately 2.6 million to 11,700 years ago) drove glacial–interglacial cycles, with glacial maxima confining species to southern refugia: the Iberian, Italian, and Balkan peninsulas, where complex topography and buffered climates preserved genetic diversity (Taberlet et al. 1998; Bennett et al. 1991; G. Hewitt 2000). These refugia also functioned as biodiversity hotspots due to their environmental stability and the persistence of deep evolutionary lineages (Médail and Quézel 1999; Myers et al. 2000; Schmitt 2007). Following glacial retreat, northward expansions produced gradients of declining genetic diversity through founder effects and serial bottlenecks (G. M. Hewitt 2004a).

This model, while widely supported across many taxa (Comes and Kadereit 1998; Schönswetter et al. 2005), has been refined with evidence for multiple extra‐Mediterranean refugia in the Alps, the Carpathians, and parts of Central and Eastern Europe (Stewart and Lister 2001; Bhagwat and Willis 2008; Schmitt and Varga 2012). Furthermore, some authors have emphasized the role of northern refugia and demonstrated that postglacial recolonization often involved admixture from several distinct source populations (Gómez and Lunt 2007), leading to a more nuanced understanding of glacial survival and postglacial range dynamics across Europe (Stewart et al. 2010; Schmitt and Varga 2012).

The Eurasian perch ( *Perca fluviatilis* L.) provides a useful focal species for studying phylogeographic structure and postglacial dynamics across its broad distributional range, which extends through temperate and northern Eurasia (Collette and Banarescu 1977; Thorpe 1977). It occupies diverse aquatic habitats, including lakes, rivers, and brackish coastal environments, and displays tolerance to a wide range of environmental gradients such as temperature, oxygen, salinity, and pH (Diehl 1992; Nilsson et al. 2004; Devlin et al. 2015; Vejřík et al. 2016; Christensen et al. 2019; Noreikiene et al. 2020). This combination makes the Eurasian perch a valuable system for exploring species‐specific responses and for investigating environmental adaptation (Ozerov et al. 2022, 2025; Noreikiene et al. 2024; López et al. 2025). Earlier genetic studies of perch, based on short mitochondrial DNA (mtDNA) fragments, microsatellites, and a small set of SNPs, have provided important initial insights into its phylogeography, historical demography, and population genetic structure. For instance, Nesbø et al. (1999) suggested the presence of four major mitochondrial lineages and admixture zones located alongside the areas where these lineages converge. More recently, Toomey et al. (2020) refined earlier findings by clarifying the patterns of genetic variability using four mtDNA regions and eight microsatellite markers. However, these studies were constrained either by limited geographic coverage or low genomic resolution. As pointed out by the authors, key regions such as the Balkans and Pannonia (Middle‐Danubian Basin) were underrepresented in the sampling of both Nesbø et al. (1999) and Toomey et al. (2020), leaving a gap that, from a phylogenomic perspective, limits the ability to draw robust evolutionary inferences. Furthermore, while microsatellites and short mtDNA fragments can reveal broad‐scale patterns, they lack the power of genome‐wide approaches to detect finer‐scale population structure (Allendorf et al. 2010; Andrews et al. 2016).

The availability of whole‐genome assemblies (Ozerov et al. 2018; Roques et al. 2020) has now positioned the Eurasian perch as a promising model for exploring ecological, geological, and evolutionary processes (Vasemägi et al. 2023). The rapid expansion of genomic resources has facilitated detailed investigations of spatiotemporal genomic patterns (Lichman et al. 2024), transcriptome responses to parasites (Taube et al. 2023), comparative genomics (Kuhl et al. 2024) and adaptation (Skovrind et al. 2023; Ozerov et al. 2022, 2025; López et al. 2025).

The objectives of this study are threefold. First, we test the utility of whole mtDNA genome resequencing for delineating the geographic spread of distinct maternal lineages, and nuclear SNP genotyping for assessing genetic diversity across an expanded sampling range. Second, we characterize global genetic diversity hotspots and assess potential latitudinal and longitudinal gradients to understand how historical climatic fluctuations and postglacial recolonization have shaped present‐day diversity patterns of perch. Finally, we explore the relationships between genetic divergence and geographic distance at both global and lineage‐specific levels to gain deeper insights into the spatial distribution of genetic differentiation. Using high‐coverage mtDNA resequencing and 3660 nuclear SNPs from 188 locations spanning the species' native range, we provide novel insights into the evolutionary history of Eurasian perch and the mechanisms shaping biodiversity.

## 2 Materials and Methods

### 2.1 Sample Collection

**FIGURE 1:**
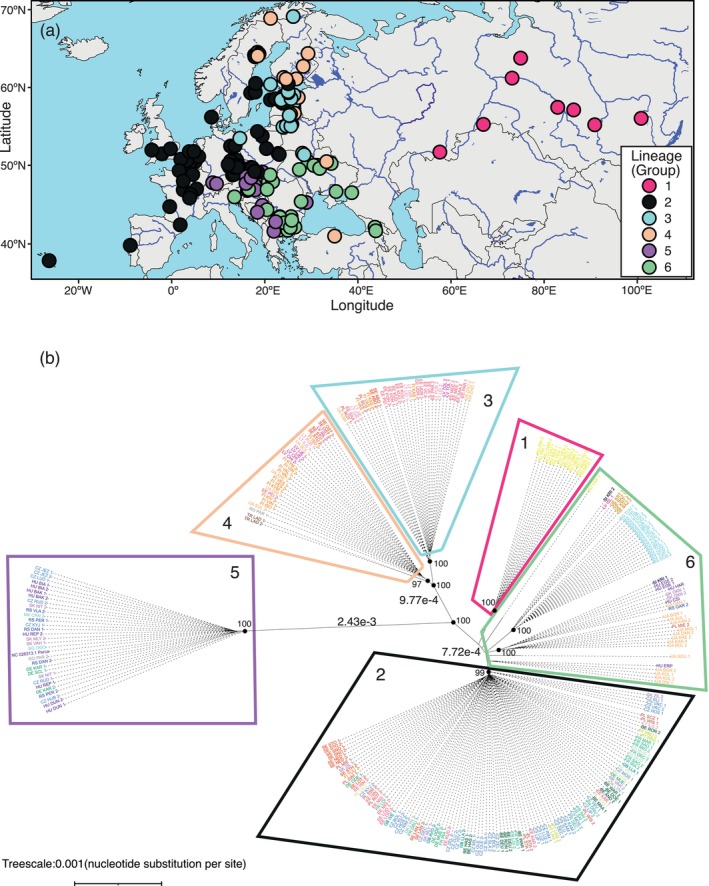
(a) Sampling locations of 269 *Perca fluviatilis* individuals from 149 sites used for mtDNA analyses. Different colors represent locations corresponding to five highly divergent mtDNA lineages (1–5) and Group 6, as shown in both (a) and the boxes in (b). (b) Maximum likelihood (ML) representation of mtDNA phylogenetic relationships among 270 *Perca fluviatilis* individuals as an unrooted tree, with the reference genome (KM410088.1) included. Tips of the phylogenetic tree, representing individuals, are colored according to their country of origin, consistent with the color scheme shown in Figure 2.

**FIGURE 2:**
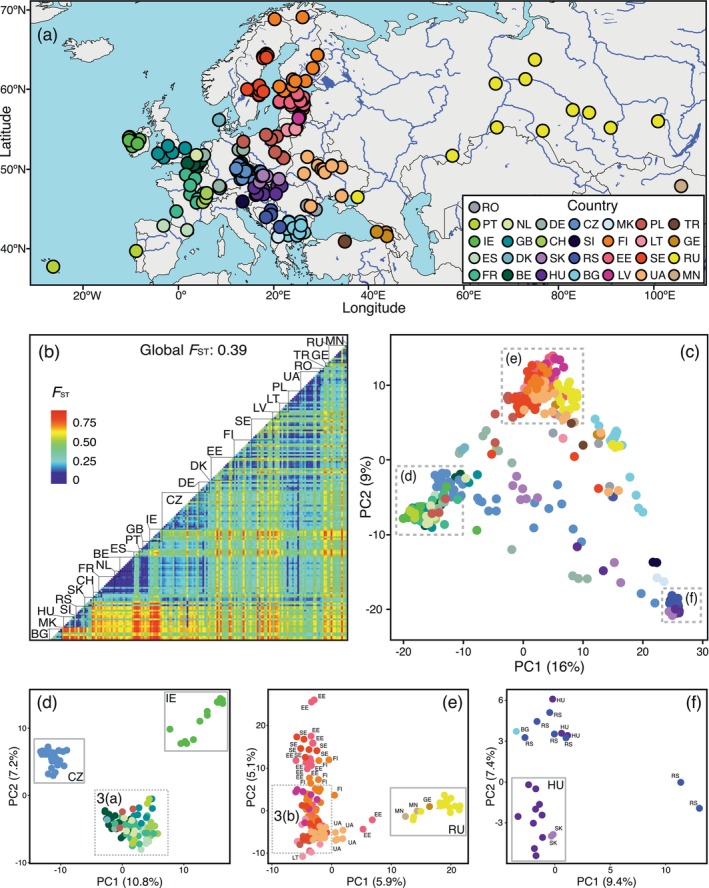
(a) Sampling locations of 363 *Perca fluviatilis* individuals from 184 sites included in the SNP array analysis. Dot colors in (a) and in the PCA plots (c–f) represent different countries. (b) Heatmap of pairwise *F*_ST_ values between populations with paired samples (npop = 169) based on the nuclear SNP array. (c) PCA plot of 363 individuals. Clusters defined as Western (d), Northern (e), and Central (f) groups are highlighted. BE, Belgium; BG, Bulgaria; CH, Switzerland; CZ, the Czech Republic; DE, Germany; DK, Denmark; EE, Estonia; ES, Spain; FI, Finland; FR, France; GB, England; GE, Georgia; HU, Hungary; IE, Ireland; LT, Lithuania; LV, Latvia; MK, North Macedonia; MN, Mongolia; NL, the Netherlands; PL, Poland; PT, Portugal; RO, Romania; RS, Serbia; RU, Russia; SE, Sweden; SI, Slovenia; SK, Slovakia; TR, Turkey; UA, Ukraine.

We analyzed a dataset comprising 382 individuals of Eurasian perch (hereafter perch) collected from 188 distinct sampling sites (Figure 1a and Table S1), spanning approximately 10,000 km from east to west (from Mongolia to Azores Islands) and approximately 3000 km from south to north (from Turkey to northern Finland). Most locations comprised two or more individuals (*n* = 179), whereas a few locations (*n* = 9) included only a single specimen. The sampling sites encompassed wild populations from a variety of environments, including lakes, rivers, ponds, canals, reservoirs, estuaries, and seas, spanning 29 countries across the species' natural distribution range (Stepien and Haponski 2015). These regions include continental Europe, the British Isles, Ireland, Northern Europe, the Caucasus, the Middle East, Central Asia, and Siberia (Figures 1a and 2a). We also included samples from perch introduced to the Iberian Peninsula and Azores (part of the Macaronesia Archipelago in the Atlantic Ocean) (Ribeiro et al. 2009; Dias et al. 2025). To further supplement the collected dataset, we incorporated 48 previously analyzed samples obtained from Finland, Estonia, Lithuania, and Sweden (Table S1) (Ozerov et al. 2022; Vasemägi et al. 2023).

The sample collection complied with national regulations and international agreements under the Nagoya Protocol. Ethical considerations during sampling followed the requirements outlined in Directive 2010/63/EU of the European Parliament and of the Council of 22 September 2010 on the protection of animals used for scientific purposes. All perch specimens were collected and euthanized in compliance with national legislation and under permits issued by local authorities (Berthelsen et al. 2025; Table S5).

### 2.2 DNA Extraction

Genomic DNA (gDNA) from 313 fish tissue samples (from various tissues, such as fins, scales, and muscle tissue) was isolated using the NucleoSpin Tissue Kit (Macherey‐Nagel GmbH) or the QIAamp 96 DNA QIAcube HT Kit (QIAGEN). An additional 69 samples of gDNA were obtained either from previous studies (Ozerov et al. 2022; Noreikiene et al. 2020, *n* = 48) or provided by collaborators (*n* = 21).

The concentration of gDNA was quantified using a NanoDrop 2000 spectrophotometer (Thermo Fisher Scientific), and DNA quality was evaluated via 1% agarose gel electrophoresis stained with ethidium bromide. Samples were then diluted to 10 ng/μL for mitochondrial DNA (mtDNA) sequencing and 30 ng/μL for nuclear SNP genotyping, using either buffer EB (for samples extracted with the NucleoSpin Tissue Kit) or buffer AE (for those isolated with the QIAcube HT Kit).

### 2.3 Analyses of Mitogenomes and Genome‐Wide Nuclear SNPs

To obtain a more comprehensive understanding of how contemporary diversity and the distribution of the species have been shaped by evolutionary history, we aimed to incorporate both whole mtDNA genome and nuclear SNP data in our analyses. Due to differences in data generation protocols for whole‐genome sequencing and SNP‐array genotyping, the number of samples included for the initial screening of the mtDNA and nuclear SNPs varied slightly (374 and 382, respectively). Consequently, we used slightly different datasets for various analyses (Table 1).

**TABLE 1:** Overview of *Perca fluviatilis* samples and datasets used for various analyses (additional details are provided in Table S1). Nind and npop indicate number of individuals and populations used for specific analyses, respectively.

Type of analysis	Data type			
	Nuclear SNP array (MultiFishSNPChip)		Nearly complete mtDNA genomes	
	nind	npop	nind	npop
Phylogenetic relationships			269	149
PCA	363	184		
Genetic diversity	363	184		
Admixture	363	184		
IBD	349	169		

Abbreviations: IBD, isolation by distance; PCA, principal component analysis.

### 2.4 Library Preparation and Whole Genome Sequencing (WGS)

Sequencing libraries for 374 mtDNA samples were prepared following the Illumina TruSeq DNA PCR‐Free Library Preparation Guide. Libraries were sequenced using a NovaSeq X Plus sequencer with 0.5 lanes of a 10B‐300 flow cell (standard paired‐end read setup of 2 × 150 bp) at the National Genomics Infrastructure (NGI), Stockholm, from 1 μg of gDNA. The average insert size was 350 bp.

### 2.5 Quality Control and Axiom SNP Genotyping Array (MultiFishSNPChip)

A total of 382 perch samples were genotyped using the MultiFishSNPChip_1.0 (DNA TRACEBACK Fisheries SNP array, FSHSTK1D), a custom high‐throughput SNP genotyping array developed for multiple fish species, including perch (Andersson et al. 2024; Vasemägi et al. 2023). Genotyping was performed by IdentiGEN Limited (Dublin, Ireland) on the Axiom platform. Allele calling and quality control were performed using thermo fisher's axiom suite software, following the manufacturer's recommended best practices. The final dataset included 3660 high‐quality polymorphic SNPs (from an initial set of 3741 SNPs) genotyped across 363 individuals from 184 locations, with an average SNP call rate of 98.32%. The final filtered SNP dataset was exported as a VCF file and subsequently converted into a genind object using the R package ‘adegenet’ v.2.1.8 (Jombart 2008; Jombart and Ahmed 2011) in R v.4.1.3 (R Core Team 2022).

### 2.6 Quality Control and Generation of Consensus mtDNA Sequences From WGS

Quality assessment of the mtDNA dataset, including 374 individuals, was conducted using fastqc (Andrews 2010). Reads were trimmed using fastp v.0.20 (Chen et al. 2018) to remove sequences < 60 bp, low‐quality reads (mean Phred score < 25), and Illumina adapters. Cleaned paired‐end reads were then aligned to the perch mitochondrial reference genome (NCBI accession: KM410088.1) using bowtie2 v.2.3.5.1 (Langmead and Salzberg 2012), with modified parameters to adjust the minimum alignment score threshold (−score‐min L, −0.3, −0.3) and the maximum fragment length for valid paired‐end reads (−X 700).

Consensus mtDNA sequences were extracted with bcftools v.1.17 (Li 2011), applying a minimum mapping quality threshold of 20 (−q 20), and converted to FASTA format using seqtk v.1.3 (Li 2013). After quality filtering, 269 nearly complete mtDNA genomes met both inclusion criteria: ≥ 95% genome coverage and a minimum average read depth (DP) of 5.

### 2.7 mtDNA Alignment and Phylogenetic Tree Construction

The mtDNA alignment of the 270 sequences (including the reference genome) was trimmed at both ends to remove 130 bp from the 5′ end and 221 bp from the 3′ end, resulting in a final alignment of 16,186 bp (97.88% of the 16,537 bp reference genome). The start and end positions were determined as the first and last sites consistently aligned across all sequences. Phylogenetic reconstruction was performed using iq‐tree2 (Minh et al. 2020), employing the modelfinder module (Kalyaanamoorthy et al. 2017) to identify the best‐fit substitution model. The TN + F + R3 model was selected based on the Bayesian Information Criterion (BIC) and used to infer a maximum likelihood (ML) consensus tree with 1000 bootstrap replicates. The final phylogenetic tree was visualized using itol v.4 (Letunic and Bork 2019).

### 2.8 Genetic Divergence

To assess genetic differentiation among populations, we calculated pairwise *F*_ST_ values for 169 populations, each represented by at least two individuals. Previous studies have shown that reliable *F*_ST_ estimates can be obtained even from small sample sizes (i.e., two individuals), provided that a sufficient number of SNPs (≥ 1500) are used (Willing et al. 2012; Nazareno et al. 2017). Allele frequencies were estimated from a *genpop* object created with the genind2genpop function, and pairwise *F*_ST_ values were then computed with the pairwise.fst function, both available in the ‘adegenet’ R package.

### 2.9 Principal Component Analysis (PCA) on Nuclear SNPs

To explore genetic relationships among individuals, we performed PCA using the dudi.pca function from the ade4 R package v.1.7–19 (Dray and Dufour 2007). PCA was conducted on two datasets: (i) all 363 genotyped individuals and (ii) a subset of 269 individuals for which overlapping mtDNA data were available. In addition, we defined several subgroups within explored groups of all individuals, for which separate *genind* objects were created and analyzed independently using PCA. Visualizations of all PCA results were generated using the factoextra R package v.1.0.7 (Kassambara and Mundt 2020).

### 2.10 Admixture Analysis on Nuclear SNPs

We employed admixture v.1.3 (Alexander et al. 2015) to estimate individual ancestry proportions in the nuclear SNP dataset (363 individuals). To minimize linkage disequilibrium (LD) effects on the inferred population structure (Falush et al. 2003; Kaeuffer et al. 2007), LD pruning was conducted using plink v.1.9 (Purcell et al. 2007) with the parameter –indep‐pairwise 1000 100 0.2, resulting in a pruned dataset of 2297 SNPs. ADMIXTURE was run across values of *K* from 1 to 30 using 500 bootstrap replicates (‐B500) and 30‐fold cross‐validation (–cv = 30).

### 2.11 Genetic Diversity Estimates Based on Nuclear SNPs

Observed heterozygosity (*H*_O_), defined as the proportion of heterozygous loci, was estimated using two datasets: (i) the full dataset comprising 363 individuals from 184 locations, and (ii) a subset of 349 individuals from 169 populations, where each population was represented by 2–4 individuals (populations represented by a single individual were excluded). *H*_O_ values were computed in PLINK v.1.9 using the –het option and cross‐validated with the allel.heterozygosity_observed function from scikit‐allel v.1.3.11 (Miles et al. 2024). *H*_O_ distributions were analyzed by country, and their associations with geographic coordinates (latitude and longitude) were assessed.

### 2.12 Isolation by Distance (IBD)

We tested for isolation by distance (IBD) to evaluate the relationship between genetic and geographic distances among populations. Geographic distances were calculated using the distVincentyEllipsoid function from the ‘geosphere’ R package v.1.5–18 (Hijmans 2022), based on transformed coordinates. Genetic distances (pairwise *F*_ST_) were computed using the pairwise.fst function from adegenet. We then applied a two‐dimensional linear model (Rousset 1997), regressing the natural logarithm of geographic distance (ln km) against *F*_ST_ / (1—*F*_ST_). Mantel tests (Pearson, 999 permutations) were performed on all 169 paired‐individual populations and separately for each mtDNA lineage group. Additionally, Mantel correlograms were generated using the mgram function from ‘ecodist’ v.2.1.3 (Goslee and Urban 2007) with a uniform interval of 100 km for both the full dataset and lineage‐specific subsets.

### 2.13 Inverse Distance Weighting (IDW) Map

To visualize spatial variation in genome‐wide genetic diversity, we constructed an inverse distance weighting (IDW) map of observed heterozygosity. Following the approach of Testo (2021), we interpolated individual *H*_O_ values using the World Geodetic System 1984 (WGS 84) projection. A spatial buffer of 250 km was applied to limit the influence of each point, and the IDW interpolation was performed with a power of 0.5 to ensure a smooth surface with moderate influence from distant samples. Based on optimization criteria, we used a minimum of 13 neighboring points for reliable surface construction.

## 3 Results

### 3.1 Phylogenetic Relationships and Distribution of mtDNA Lineages

Based on 269 nearly complete mtDNA genomes, we identified five deeply divergent monophyletic lineages (1–5), each supported by strong bootstrap support for the clades (Figure 1b). Furthermore, the broader sample coverage of our study revealed a group of haplotypes located between lineages 1 and 2 occurring in Southeastern Europe (Georgia, Ukraine, Bulgaria, Hungary, Slovakia, and Slovenia). We denote this group tentatively as Group 6 because of its clear geographical separation despite its non‐monophyletic nature.

The major mtDNA lineages are separated by long internal branches, indicating substantial evolutionary divergence and supporting their deep‐rooted origin. In particular, Lineage 5 exhibits an early split in the phylogeny, as evidenced by pronounced internal branch lengths that clearly distinguish it from all other lineages. This topology is more consistent with a basal divergence event than with a recent or hierarchically nested subdivision within the principal radiation. In turn, Group 6 diverges from the evolutionary framework observed in the other mtDNA lineages, reflecting a more recent evolutionary divergence.

The geographic ranges of each lineage are as follows: Lineage 1 is exclusively represented by populations sampled from the Ural and Siberian regions of Russia (Figure 1a). Lineage 2 is primarily composed of samples from Western Europe, with a subset of individuals reaching the Baltic Sea basin. Lineage 3 is restricted to samples from the Baltic Sea region. Similarly, Lineage 4 predominantly consists of samples from the Baltic Sea basin, occurring northward and eastward relative to Lineage 3, with a few cases reaching the Black Sea basin. Lineage 5 is distributed across Central Europe (Pannonian Basin and surrounding areas, Danube basin) and the Balkans. Lastly, Group 6 is found in the Black Sea region, with partial representation in the Balkans and Central Europe.

### 3.2 Genetic Divergence and Structuring

Genome‐wide nuclear SNPs revealed substantial divergence among the studied perch populations, with a global *F*_ST_ value of 0.39, reflecting a high level of structuring (Figure 2b and Figure S1b; Table S2). The most highly divergent groups (mean *F*_ST_ = 0.43–0.48; Figure S4) were observed in populations from Hungary (HU), Bulgaria (BG), Serbia (RS), and Ireland (IE). Notably, many populations exhibited significant divergence regardless of geographic proximity, indicating a strong influence of genetic drift and isolation. The lowest *F*_ST_ values were observed within the Baltic countries (mean *F*_ST_ = 0.16–0.19): Sweden (SE), Estonia (EE), Finland (FI), and Poland (PL), as well as in Ukraine (UA) and Germany (DE). Pairwise *F*ST values among populations from these countries were considerably lower compared to those within countries in Central Europe and the Balkans (Figure S4).

PCA of the nuclear SNP dataset identified three broadly defined genetic groups (Figure 2c). The first group, located on the leftmost side of the PC1 axis, comprises individuals from Western Europe, including samples from Ireland, Portugal, Spain, Switzerland, Germany, England, Belgium, France, and the Czech Republic. The second group, located on the uppermost side of the PC2 axis, primarily includes individuals from the Baltic Sea region (Poland, Sweden, Finland, Estonia, Latvia, and Lithuania), with some divergence from samples from Ukraine, Russia, Mongolia, and Georgia. The third group, located on the rightmost side of PC1, consists mainly of individuals from Hungary, Bulgaria, and Serbia (Central and Southeastern Europe).

**FIGURE 3:**
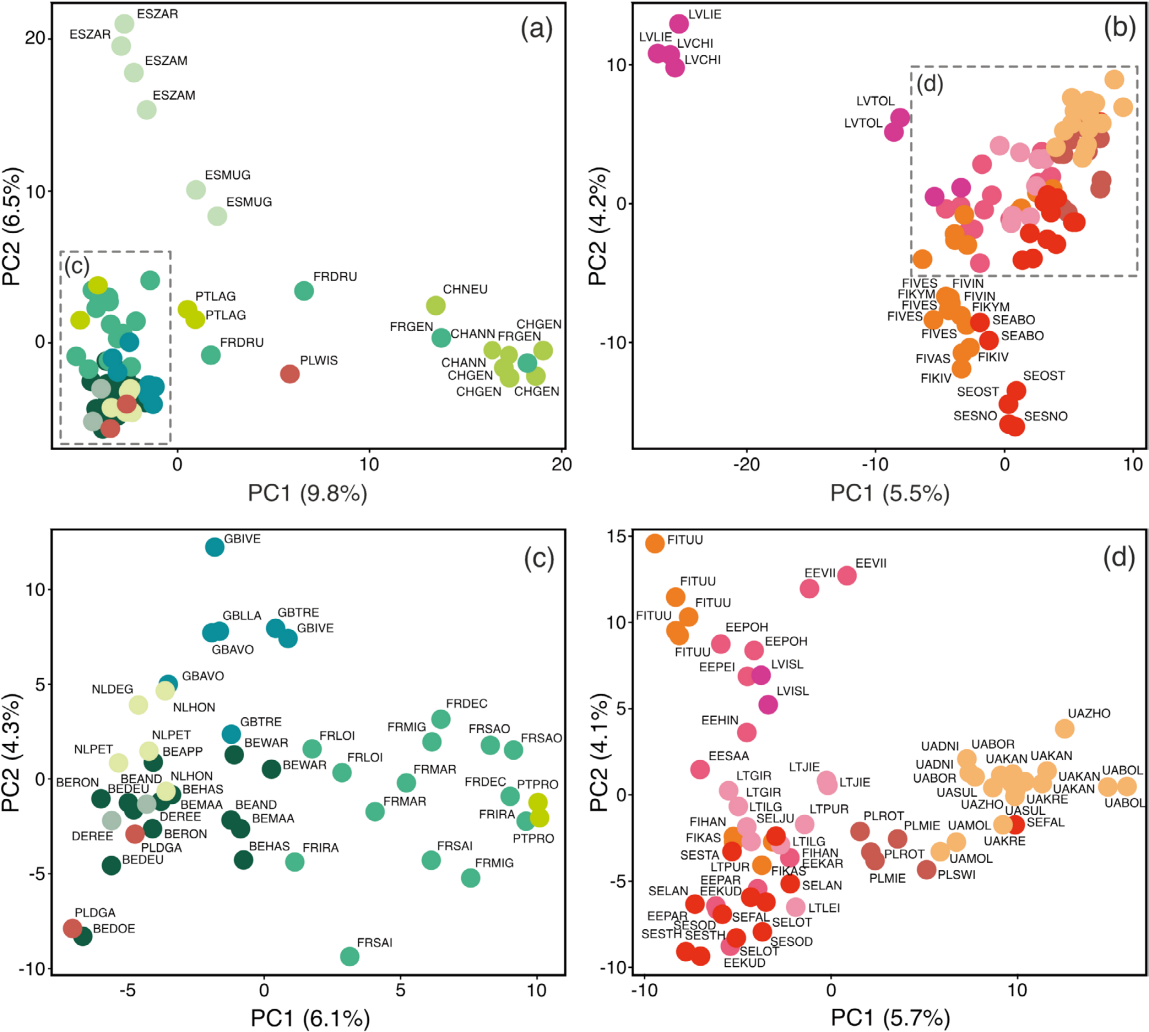
Principal component analysis (PCA) plots of *Perca fluviatilis* samples within the Western (a) and Northern (b) groups, with subsequent magnification of densely clustered regions for subsets of the Western (c, d) Northern groups, respectively.

Figure 2d–f provide more detailed PCA results of groups 1, 2, and 3, respectively. Figure 2d highlights Western European samples, emphasizing the genetic divergence of Irish and Czech samples from the rest. Further analysis of this group (Figure 3a) also reveals the isolation of samples from Spain and Lake Geneva (France and Switzerland) compared to individuals from the rest of the countries (Portugal, Germany, Spain, the Netherlands, England, France, Belgium, and Poland). Finally, PCA of the densest subgroup within this group (Figure 3c) shows a clear separation along PC1 between populations from Belgium, Germany, and the Netherlands on one side, and France and Portugal on the other, while samples from England remain the most distinct along PC2.

Figure 2e shows a detailed view of individuals mainly distributed in the Baltics, while also highlighting the separation of Russian samples, a single specimen from Georgia, and two from Mongolia. Further differentiation (Figure 3b) reveals distinct Latvian populations and a split between Finnish and Swedish samples from eight locations and the rest of the group. Zooming into the densest cluster (Figure 3d) shows a clear separation along PC1 between Ukrainian populations from the Dnipro River and its tributaries and those from Sweden and Finland, with Polish and Lithuanian populations in intermediate positions.

The most genetically distinct group (Figure 2f) comprises primarily Lineage 5 and Group 6 individuals, with a core of samples from Central Europe, particularly Hungary and Slovakia. Overlaying mitochondrial lineages on the PCA (Figure S1a) reveals a consistent pattern: Western European individuals cluster together as mtDNA Lineage 2, also present in Northern Europe, while the Northern group includes Lineages 1, 2, 3, and 4, and the Southeastern European cluster is dominated by Lineage 5 and Group 6.

**FIGURE 4:**
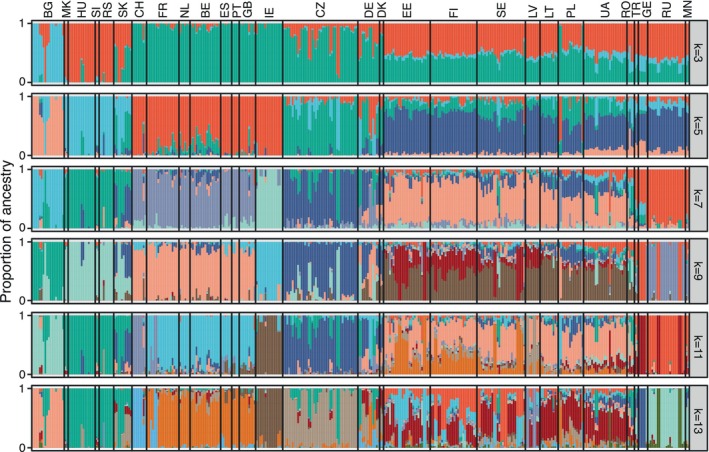
Ancestry inference of 363 *Perca fluviatilis* individuals based on 2297 nuclear SNPs, performed using ADMIXTURE with varying numbers of clusters (*K* = 3, 5, 7, 9, 11, 13).

To further investigate genetic structure and ancestry proportions across all populations, we performed an ADMIXTURE analysis (Figure 4 and Figure S2). Cross‐validation error identified *K* = 13 as the optimal clustering level, revealing strong genetic divergence by country and clear regional groupings. At *K* = 2, Southeastern and Central European populations (Bulgaria, North Macedonia, Serbia, Slovenia, and Hungary) separate from Western European populations (Switzerland, France, the Netherlands, Belgium, Spain, Portugal, Great Britain, and Ireland), while other samples show varying levels of admixture. As the number of clusters increases (*K* = 3 to *K* = 5), genetic structure within Europe becomes more pronounced: at *K* = 3, Bulgarian specimens separate from other Balkan populations; at *K* = 4, populations from Fennoscandia and the Baltic region (Sweden, Finland, Estonia, Latvia, and Lithuania) form a shared ancestry group; and at *K* = 5, the Czech Republic emerges as a distinct genetic group, diverging from other Central European populations.

With further increases in K, additional unique clusters appear. At *K* = 6, individuals from Russia and Mongolia form a group separate from those in Ukraine and Romania; at K = 7, Irish populations split from Great Britain; and at *K* = 8, Polish and Lithuanian populations separate from Estonia, Finland, and Sweden (Figure S2). At *K* = 9, Georgian perch form their own cluster, distinct from Turkish and Russian samples, and at higher values (*K* = 10–13), additional smaller clusters emerge (e.g., Latvia at *K* = 10, Switzerland at *K* = 11). Beyond *K* = 11, ADMIXTURE no longer identifies country‐level clusters but instead highlights divergence at the level of distinct populations.

### 3.3 Spatial Distribution of Nuclear Diversity

**FIGURE 5:**
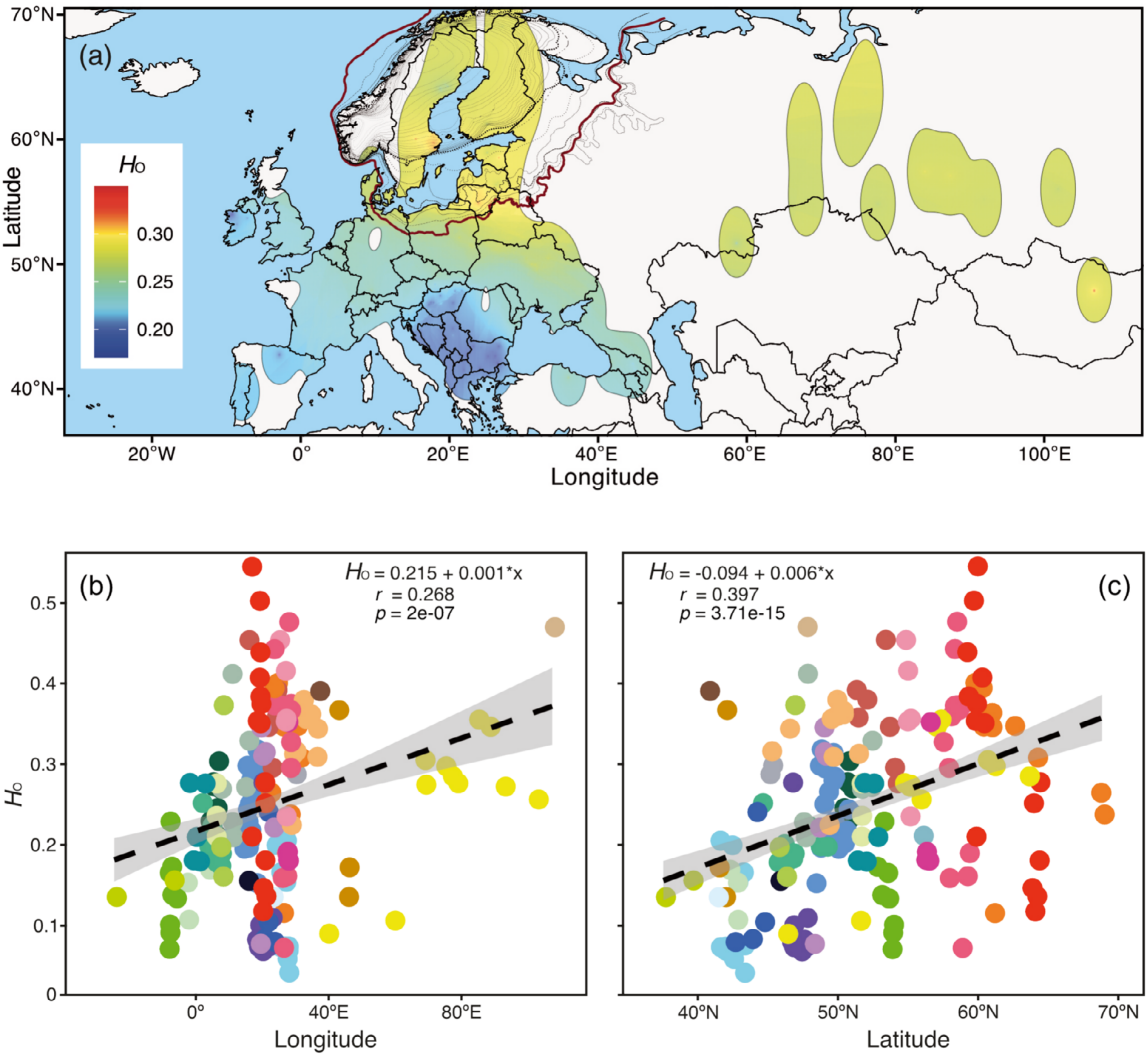
(a) Inverse‐distance weighted (IDW) plot of heterozygosity observed (*H*_O_) within 184 populations of *Perca fluviatilis* , shown in the WGS84 (World Geodetic System 1984) projection. In this ellipsoidal projection, longitude spacing remains constant while latitude varies with position, resulting in a variable radius for the buffered zones (250 km) around sampling points depending on latitude. Isochrones represent the retreat of the Fennoscandian Ice Sheet after the Last Glacial Maximum (LGM) (Stroeven et al. 2016). The outermost ice margin during the local LGM (ca. 20,000 years ago) is highlighted in dark red, while the Younger Dryas margin (ca. 12,000 years ago) is shown as a bold dashed line. The deglaciated zone between these two margins is segmented by narrow dashed isochrones marking ca. 1000‐year intervals (19,000–12,700 years ago), whereas the zone enclosed within the Younger Dryas margin contains dotted isochrones marking ca. 100‐year intervals (11,600–9700 years ago). Linear regression plots depict relationships between observed heterozygosity (*H*_O_) and longitude (b) and latitude (c), respectively. Colors indicate countries, corresponding to those in Figure 2.

The IDW interpolation map revealed a pronounced contrast in diversity between Southern and Northern Europe, with the Scandinavian and Baltic regions representing hotspots for genetic diversity (Figure 5a). High diversity was also observed among the Mongolian (mean *H*_O_ = 0.46) and Siberian samples (mean *H*_O_ = 0.25), although sampling density in those regions was much lower compared to that in Europe. The lowest levels of genetic diversity were observed in Southern Europe, particularly in the Balkan Peninsula (e.g., mean *H*_O_ = 0.10 in Bulgaria) and the Iberian Peninsula (mean *H*_O_ = 0.14 for both Portugal and Spain), as well as in Ireland (mean *H*_O_ = 0.13; Figure S1c). Consistent with the IDW results, linear regression analyses revealed a highly significant increase in genetic diversity from south to north and from west to east, with latitude explaining more variation than longitude (15% vs. 7%, respectively; Figure 5b,c). To ensure that the observed longitudinal pattern was not disproportionately influenced by the few easternmost populations, we repeated the analysis after excluding samples east of 50° E. The pattern remained similar, with the regression for this subset yielding *H*_O_ = 0.002 * Longitude +0.201 (*r* = 0.252, *p* = 8.36e‐4), compared with *H*_O_ = 0.001 * Longitude +0.215 (*r* = 0.268, *p* = 2e‐07) for the full dataset.

### 3.4 Isolation by Distance

**FIGURE 6:**
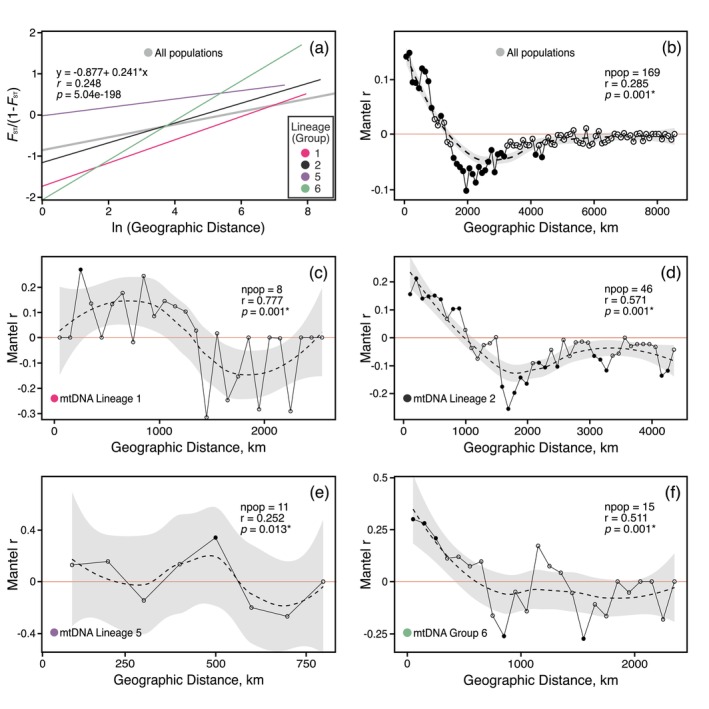
Isolation by Distance (IBD) linear model plot constructed for 169 *Perca fluviatilis* populations as well as mtDNA lineages 1, 2, 5, and Group 6 (a), alongside the corresponding Mantel correlogram for the entire dataset (b). Panels (c–f) show Mantel correlograms for mtDNA lineages 1, 2, 5, and Group 6, respectively.

The results of the IBD analysis, conducted on 169 locations, revealed a moderate but highly significant correlation (Pearson's *r* = 0.248, *p* = 5.04e‐198) between genetic divergence and geographic distance (Figure 6a). Moran's correlograms provided further insights into the spatial patterns of genetic variation. We observed the highest correlations between pairwise *F*_ST_ and geographic distance (Mantel *r* = 0.285, *p* = 0.01) at the smallest geographical scale (0–200 km), which decreased sharply at larger distances (Figure 6b).

We subsequently tested the relationship between genetic and geographic distances separately for six distinct mtDNA groups to evaluate whether different postglacial colonization routes have potentially shaped present‐day IBD patterns. We observed significant IBD patterns for Lineages 1, 2, 5, and Group 6 (Figure 6a), but not for Lineages 3 and 4 (Figure S3a,c). However, the Mantel correlogram showed significant positive correlations between pairwise *F*ST and geographic distance at the smallest geographical scale only for mtDNA Lineage 2 and Group 6 (Figure 6d,f), while for the other lineages, the Mantel r across short distance classes was not significant (Figure 6c,e).

## 4 Discussion

Our study reveals several key findings regarding the phylogeography and genetic diversity of perch across its native range. First, the Baltic Sea region emerges as a contemporary hotspot of genetic diversity, confirming previous findings but now contextualized by genome‐wide markers and broader geographic coverage. Second, we observe a clear longitudinal decline in genetic variability, with notably reduced diversity in Western European populations. Third, in addition to confirming the geographical distribution of mtDNA lineages 1–5, we identified a previously uncharacterized group of mitochondrial haplotypes (Group 6) occurring between lineages 1 and 2. Finally, our analyses revealed that the Danube basin harbors the most divergent mitochondrial lineage (Lineage 5), which exhibits a broader geographical distribution than previously recognized.

### 4.1 Baltic Sea Basin as a Northern Biodiversity Hotspot

Earlier research suggested the Baltic Sea region as a secondary contact zone for perch, characterized by the coexistence and introgression of multiple divergent mitochondrial lineages (Nesbø et al. 1999). This was further supported by Toomey et al. (2020), who found high mitochondrial haplotype diversity in perch populations across the Baltic region, particularly in the east, consistent with secondary contact and admixture among divergent postglacial lineages. By assembling the most comprehensive genome‐wide *P. fluviatilis* dataset to date, consisting of nearly complete mitochondrial genomes and genome‐wide nuclear SNPs, we not only confirmed the presence of multiple mitochondrial lineages in the Baltic Sea but also revealed elevated heterozygosity and widespread admixture signals, providing strong evidence for introgression among lineages. Additionally, we identified a pronounced latitudinal gradient in nuclear genetic diversity, highlighting the Baltic Sea as a contemporary biodiversity hotspot.

These findings challenge the traditional “southern richness and northern purity” paradigm (G. M. Hewitt 2004a), which posits that genetic diversity decreases with increasing latitude due to serial founder effects during postglacial recolonization (G. Hewitt 2000). Instead, our results resemble patterns observed in other northern fishes, such as the three‐spined stickleback ( *Gasterosteus aculeatus* ) and European whitefish ( *Coregonus lavaretus* ). These species have been shown to maintain relatively high levels of genetic diversity, linked to either multiple colonization events from distinct glacial refugia or secondary contact between previously isolated lineages following ice retreat (Coll‐Costa et al. 2024; Østbye et al. 2006). Such scenarios suggest that northern regions can retain or regain substantial genetic diversity when colonization is not strictly unidirectional or does not follow a stepwise pattern.

Toomey et al. (2020) reported increased genetic variability in perch populations across Eastern Europe, Fennoscandia, and Central Europe, deviating from the pattern observed in many freshwater taxa that typically exhibit diversity hotspots in southern refugia (Durand et al. 1999; Gum et al. 2005; but see Kotlik and Berrebi 2001). In our study, freshwater habitats within the Baltic Sea area emerged as key hotspots of genetic diversity. This region exhibits the highest levels of heterozygosity (Figure 5a) and serves as a contact zone for divergent mitochondrial lineages (2, 3, and 4), strongly suggesting that the observed patterns reflect genuine biological processes, such as historical admixture and secondary contact, rather than methodological artifacts (see below). This interpretation is further supported by the admixture analyses, where Baltic populations consistently display mixed ancestry across the full range of inferred ancestral clusters (*K* = 2–13; Figure 4 and Figure S2). The stable proportions of shared ancestry observed across K‐values point to long‐term admixture among lineages rather than recent or ephemeral gene flow.

### 4.2 Distribution of mtDNA Lineages and Patterns of Recolonization

The phylogenomic structure of perch revealed deep historical divergences, with multiple mtDNA lineages likely originating from isolated, distinct glacial refugia. Lineage 5 is particularly notable for its long‐term evolutionary distinctiveness among perch lineages, as evidenced by its high mitochondrial divergence (0.0024 substitutions per site) on the ML phylogenetic tree (Figure 1a) and its strong separation in the nuclear SNP‐array PCA (Figure S1a). This deep branching was initially described by Nesbø et al. (1999) and has since been confirmed by divergence time estimates, dating the split to approximately 1.4 million years ago (95% highest posterior density interval: 0.73–2.27 million years ago; Lichman et al. 2024), thereby establishing Lineage 5 as the most divergent in perch. Confined primarily to the Central Balkans and the Danube basin, both the genetic distinctiveness and distribution range of this lineage corroborate previous findings (Nesbø et al. 1999; Vasemägi et al. 2023; Lichman et al. 2024) and parallel patterns observed in other freshwater taxa such as *Barbus*, *Squalius*, and *Rutilus* species complexes, where the mountainous regions around the Danube served both as refugia and centers of speciation (Schmitt and Varga 2012; Oikonomou et al. 2014).

In contrast, lineages 3 and 4 display a broader geographic spread, most likely reflecting recolonization through extensive hydrological connectivity following glacial retreat. The spread of Lineage 4 from the Black Sea to Northern Europe through river systems such as the Dnipro and Neva supports the hypothesis of a freshwater corridor during late‐glacial periods (Bahr et al. 2005), echoing findings in European bullhead ( *Cottus gobio* ; Kontula and Väinölä 2001). Furthermore, the co‐occurrence and admixture of lineages 2, 3, and 4 in the Baltic region point to secondary contact zones and complex colonization scenarios involving multiple source populations, as previously suggested for European whitefish (Østbye et al. 2005).

Mitochondrial Lineage 2, widely distributed in Western and Northern Europe, likely originates from a western European refugium as proposed by Nesbø et al. (1999). This hypothesis is consistent with postglacial colonization patterns observed in other freshwater and terrestrial taxa that persisted during the Last Glacial Maximum (LGM) in refugia located in southwestern Europe, particularly in the Iberian Peninsula (Gómez and Lunt 2007) and southwestern France (Sommer and Nadachowski 2006). Such areas have been identified as key refugial zones for freshwater fishes (Costedoat and Gilles 2009), as well as for terrestrial species such as the ocellated lizard (*Lacerta lepida*; Miraldo et al. 2011) and the common wall lizard ( *Podarcis muralis* ; Salvi et al. 2013), providing conditions that enabled the long‐term survival and development of regionally endemic populations.

Mitochondrial Lineage 1 comprises populations distributed across Western Siberia, spanning from the Caspian Sea basin to Lake Baikal. Earlier findings revealed genetic and phylogeographic evidence suggesting that these populations may trace their ancestry to glacial refugia that persisted through the penultimate interglacial period (approximately 150,000 years ago; Mangerud et al. 2004). These refugia likely served as critical sanctuaries, enabling the persistence of perch populations during Pleistocene climatic oscillations and contributing to the distinct evolutionary history observed within this lineage (Lichman et al. 2024).

Finally, we observed a geographically structured group of mtDNA haplotypes (Group 6) distributed across regions west, north, and east of the Black Sea. Distinct clades within this lineage primarily belong to the drainage networks of the Danube and Dnipro rivers. Thus, the phylogenetic structure closely mirrors the major hydrological systems of the Black Sea basin, suggesting that watershed boundaries have played a significant role in shaping genetic divergence. Within this framework, individuals from Bulgaria and Georgia each form monophyletic subclades, which are sister to each other (Figure 1b), pointing to a shared postglacial recolonization from the Black Sea basin. In this context, the mountain ranges to the west (Carpathians and Balkans) and east (Caucasus) most likely acted as barriers, limiting expansion and facilitating regional genetic differentiation.

Interestingly, the geographic distribution of mitochondrial lineages in perch closely matches the major biogeographic regions (BRs) proposed by Reyjol et al. (2007), which were identified through hierarchical cluster analysis of freshwater fish assemblages across 406 hydrographical networks in 233 species, reflecting historical and ecological patterns in European freshwater communities. Specifically, mtDNA Lineage 2 is mainly found within BR7 (Western Europe), Lineage 3 corresponds to BR6 (Baltic Sea basin), Lineage 4 aligns with BR5 (Northeast Europe), and Group 6 is largely restricted to BR4 (Black Sea region). This suggests that these BRs capture significant evolutionary breaks shaped by postglacial recolonization and basin isolation. However, mtDNA Lineage 5, which is both the most genetically distinct and confined to the Danube and Pannonian regions, is not completely captured by any of these predefined BR units. Instead, our findings suggest that the Pannonian Basin may represent a distinct biogeographic unit, reinforcing its role as a long‐term glacial refugium. For instance, endemic taxa such as the gastropod *Melanopsis parreyssi* and the amphibian Danube newt ( *Triturus dobrogicus* ) exhibit similar biogeographic patterns, underscoring the Pannonian Basin's role as a persistent refugium and speciation hotspot (Sümegi et al. 2018; Vörös et al. 2016). Hence, the observed divergence highlights that fine‐scale phylogeographic structure can reveal cryptic diversity and isolated lineages missed by regional biogeographic categorizations.

### 4.3 Population Structuring at Different Geographical Scales

PCA and ADMIXTURE analyses revealed pronounced population structuring of perch across the study area, indicative of restricted gene flow and isolation, further supported by strong genetic differentiation (global *F*_ST_ = 0.39, Figure 2b and Figure S1b). Distinct genetic clusters were identified, corresponding to three major geographic regions: (i) Western Europe, (ii) Northern Europe (Baltic‐Fennoscandia), and (iii) Southeastern‐Central Europe (Danube‐Balkans). This broad spatial pattern likely reflects postglacial recolonization dynamics, admixture, and persistent geographic barriers.

Our findings also highlight the importance of river drainage systems in shaping fine‐scale genetic structure. This pattern is particularly pronounced in Central Europe, including Slovakia, the Czech Republic, Germany, and Poland, where individuals within the same hydrographical basins, such as the Danube, Elbe, Rhine, and Oder, consistently exhibit shared ancestry. Here, genetic similarity often aligns more closely with drainage affiliation, highlighting the historical influence of river systems on gene flow and population connectivity. In addition, individuals from Bulgaria, Hungary, and Serbia within the Danube River basin show a clear signal of shared genetic ancestry, suggesting long‐standing connectivity along this major waterway. These populations also exhibit higher heterozygosity compared to nearby populations from more isolated drainages (Figure S1c; Tables S1 and S3), indicating that the Danube has likely functioned as a key connectivity pathway facilitating gene flow. Similar drainage‐based structuring is observed within the Dnipro and Rhine basins, where genetic clustering reflects the organization of river networks.

These results are consistent with and extend the findings of Nesbø et al. (1999), who reported strong population differentiation in perch structured by both drainage basins and national boundaries. Furthermore, ADMIXTURE analyses (*K* = 2–13), show a hierarchical genetic structure broad regional divisions appear at low *K* (i.e., between Western Europe and the rest of Eurasia at *K* = 2), whereas higher *K* values reveal distinct national‐level patterns (e.g., Bulgaria at *K* = 3, the Czech Republic at *K* = 5, Ireland at *K* = 7, and Switzerland at *K* = 11). Thus, both drainage systems and, to some extent, country boundaries and major river basins contribute to perch population structuring across Europe.

Populations from Western Siberia and Mongolia were genetically distinct from those in Europe, as evidenced by their consistent separation in ADMIXTURE analysis and PCA (Figures 2f and 4; Figure S2). These samples belong to a unique mitochondrial lineage absent in European populations, suggesting long‐term isolation and divergence likely linked to Siberian glacial refugia. However, the ADMIXTURE results also indicate some potential historical connection. Additionally, both Russian and Mongolian populations exhibit elevated heterozygosity, which may indicate historically large effective population sizes (Lichman et al. 2024).

The spatial genetic structure observed in Central Europe mirrors the patterns in zander ( *Sander lucioperca* ) and northern pike ( *Esox lucius* ), where population structure reflects historical drainage systems and anthropogenic fragmentation (Eschbach et al. 2014; Eschbach et al. 2021). In both cases, genetically distinct clusters corresponded to major catchments and were further shaped by habitat isolation. Similar genetic subdivisions driven by limited dispersal and long‐term landscape barriers have also been reported in other widespread freshwater fishes, including whitefish and loaches (*Cobitis* spp.; Avise 2000; Bernatchez and Wilson 1998).

Additionally, demographic processes such as founder effects and historical population bottlenecks, which reduce effective population size and genetic diversity, have likely contributed to the patterns of genetic differentiation observed among populations. Of particular interest is the genetic distinctiveness of perch populations in Ireland, which was strongly supported by both PCA and ADMIXTURE analyses (at *K* = 7). These findings challenge previous suggestions of predominantly anthropogenic introductions of freshwater fauna and instead support the hypothesis of strong genetic separation from the British Isles and potential natural colonization (Carlsson et al. 2014).

### 4.4 Isolation by Distance (IBD)

IBD analysis across 169 populations (Table S4) revealed a moderate (Pearson's *r* = 0.248), but statistically highly significant (*p* = 5.04e‐198) correlation between genetic and geographic distances, indicating that genetic similarity generally decreases as geographic distance increases (Figure 6a). A Mantel correlogram showed that the strongest genetic autocorrelation occurs within the shortest distance classes, usually up to 100 km (Figure 6b). This pattern reflects the limited dispersal capabilities typical of perch and many other freshwater fishes, which contribute to pronounced genetic structuring at fine geographic scales (Schmidt and Schaefer 2018; Washburn et al. 2020).

When analyzing distinct mtDNA lineages separately, significant IBD patterns were detected in three of the five lineages (1, 2, 5), and Group 6, suggesting lineage‐specific structuring patterns shaped by postglacial recolonization. Alternatively, the lack of significant IBD in Lineage 3 and 4 could be attributed to limited statistical power due to small sample sizes. At the same time, it is important to consider the ecological heterogeneity within our dataset: northern populations were predominantly collected from lakes, whereas southern populations originated primarily from rivers and reservoirs. This variation in habitat type, combined with the dendritic nature of river networks and physical catchment divides, may have contributed to the observed differences in IBD (Hughes et al. 2009; Wofford et al. 2005).

Further insights were provided by Mantel correlogram analyses, which revealed that only Lineage 2 and Group 6 exhibited strong positive autocorrelation at the smallest spatial scale (100–200 km). Such fine‐scale signals of restricted gene flow have also been documented in other European freshwater fishes, for example, in roach ( *Rutilus rutilus* ), European grayling ( *Thymallus thymallus* ), and European whitefish, where differing recolonization routes after glacial retreat generated regionally confined genetic clusters with distinct levels of connectivity (Bernatchez and Wilson 1998; Weiss et al. 2018; Crookes and Shaw 2016).

Overall, our results reinforce the notion that postglacial history exerts a broad influence on contemporary genetic landscapes in freshwater species. The observed decay of genetic similarity over short distances highlights the interaction between historical demographic processes and ongoing ecological constraints, such as riverine topology and limited dispersal, in maintaining fine‐scale genetic differentiation. Comparable patterns in grayling (Swatdipong et al. 2009), northern pike (Nordahl et al. 2019), and whitefish complexes (Bernatchez and Wilson 1998) point to the generality of these biogeographic and evolutionary processes across Europe's freshwater fauna.

### 4.5 Limitations

Ascertainment bias, which occurs when SNPs are discovered in a restricted subset of individuals and therefore do not represent genome‐wide variation, is a critical factor to consider in SNP‐chip–based population genetic studies (Albrechtsen et al. 2010). This bias can arise when SNPs are initially identified in a limited or unrepresentative subset of populations, potentially skewing the depiction of genetic variation across broader geographic scales (Lachance and Tishkoff 2013). Specifically, common variants from the discovery panel tend to be overrepresented, whereas rare or population‐specific variants may be underrepresented or missed entirely. Such bias can affect estimates of genetic diversity and differentiation, potentially leading to misleading interpretations of demographic history or gene flow (Albrechtsen et al. 2010). In our study, the SNP discovery panel comprised 42 whole genomes from 21 lakes in northern Europe and western Siberia (Vasemägi et al. 2023), which may have introduced some bias. However, the increased genetic diversity of perch around the Baltic Sea area has been independently reported by Toomey et al. (2020) based on eight microsatellite markers. Altogether, this convergence of findings supports the conclusion that the observed patterns reflect genuine biological processes rather than artifacts of ascertainment bias.

While the limited sample size per population represents an important caveat, both simulation‐based and empirical studies indicate that reliable estimates of genetic differentiation can be achieved when the number of loci is sufficiently large. For example, Nazareno et al. (2017) demonstrated that sampling as few as two individuals per population can yield robust *F*_ST_ estimates when genome‐wide SNP datasets are used. Similarly, Willing et al. (2012) showed through simulations that even two to four individuals per population are generally sufficient for reliable inference, provided that thousands of SNPs (≥ 1500) are included. In our study, approximately 3600 SNPs were analyzed, substantially exceeding this suggested threshold. In this regard, our sampling strategy represents a deliberate trade‐off between within‐population sampling depth and the extensive geographic coverage required to characterize broad‐scale phylogeographic structure. By sampling two individuals per locality, we ensured standardized minimal representation while retaining the capacity to compare patterns across a very large number of populations.

We used straight‐line geographic distances (Euclidean) to quantify spatial separation among populations. This choice was motivated by the exceptionally broad, pan‐continental scale of the study, which spans multiple inland drainage basins, coastal systems, and marine connections. Applying hydrological distance measures consistently across such a large and heterogeneous area is challenging, particularly because river systems and hydrological connectivity have changed substantially during glacial–interglacial cycles. As a result, present‐day watercourse configurations may not accurately reflect historical dispersal pathways relevant to the observed genetic structure.

## 5 Conclusions

In summary, the phylogeography and genetic diversity of perch across Europe reflect the combined influence of historical glacial refugia, postglacial recolonization, secondary contact, and contemporary dispersal limitations. The Baltic Sea emerges as a hotspot of diversity, while population structure is shaped by river networks, geographic barriers, and lineage‐specific processes. Together, these results highlight the complex interplay of historical and ecological factors in generating fine‐scale genetic differentiation and regional biodiversity patterns across the species' native range. By linking historical refugia with present‐day dispersal constraints, this work highlights the value of a genomic perspective for guiding conservation and management strategies.

## Author Contributions

**Vitalii Lichman:** conceptualization (equal), formal analysis (lead), investigation (lead), methodology (supporting), project administration (supporting), software (lead), visualization (lead), writing – original draft (lead), writing – review and editing (supporting). **Mikhail Ozerov:** conceptualization (equal), software (equal), writing – review and editing (equal). **María‐Eugenia López:** conceptualization (equal), methodology (equal), software (supporting), writing – review and editing (equal). **Kristina Noreikiene:** conceptualization (equal), project administration (equal), supervision (supporting), writing – review and editing (supporting). **Siim Kahar:** conceptualization (supporting), resources (lead), writing – review and editing (supporting). **Lilian Pukk:** conceptualization (equal), project administration (equal), writing – original draft (supporting), writing – review and editing (equal). **Oksana Burimski:** conceptualization (supporting), formal analysis (supporting), methodology (supporting), writing – original draft (supporting), writing – review and editing (supporting). **Bella Japoshvili:** resources (lead), writing – review and editing (supporting). **Dijana Blazhekovikj‐Dimovska:** resources (lead), writing – review and editing (supporting). **Dmitry Lajus:** resources (lead), writing – review and editing (supporting). **Dušan Nikolić:** resources (lead), writing – original draft (supporting), writing – review and editing (supporting). **Filipe Ribeiro:** resources (lead). **Tatyana Gebauer:** resources (equal), validation (supporting), writing – original draft (supporting), writing – review and editing (supporting). **Jan Kouřil:** resources (supporting). **Jiří Peterka:** resources (equal), writing – review and editing (supporting). **Petr Blabolil:** resources (equal), writing – review and editing (supporting). **Martin Čech:** resources (supporting), writing – review and editing (supporting). **Tomáš Jůza:** resources (supporting). **Jan Kubečka:** resources (supporting), writing – review and editing (supporting). **Milan Muška:** resources (supporting). **Marek Šmejkal:** resources (equal), writing – review and editing (supporting). **Mojmír Vašek:** resources (equal), writing – review and editing (supporting). **Kimmo Kahilainen:** conceptualization (supporting), resources (lead), writing – original draft (equal), writing – review and editing (equal). **Linas Ložys:** resources (supporting), writing – review and editing (supporting). **Jens Carlsson:** resources (equal), writing – review and editing (supporting). **William Corcoran:** resources (equal). **Özgen Yilmaz:** resources (equal), writing – original draft (supporting), writing – review and editing (supporting). **Peter Eklöv:** resources (equal), writing – original draft (supporting), writing – review and editing (supporting). **Péter Takács:** resources (equal), writing – original draft (supporting), writing – review and editing (supporting). **Bálint Bánó:** resources (supporting). **Réka Pallos:** resources (supporting). **Stefan Kazakov:** resources (equal). **Luchezar Pehlivanov:** resources (supporting). **Thomas Lecocq:** methodology (supporting), resources (equal), writing – original draft (supporting), writing – review and editing (supporting). **Sophie Lambert:** resources (supporting). **Torben Lauridsen:** resources (equal). **Andreas Berthelsen:** resources (supporting), writing – original draft (supporting), writing – review and editing (supporting). **Pedro Raposeiro:** resources (supporting). **Hugo Verreycken:** resources (supporting). **Robert Britton:** resources (supporting). **Jost Borcheling:** resources (supporting). **Yuliia Kutsokon:** resources (equal). **Oleksandr Didenko:** resources (supporting). **Pavel Jurajda:** resources (supporting). **Rafael Miranda:** resources (supporting). **Riho Gross:** conceptualization (equal), funding acquisition (equal), project administration (equal), supervision (equal). **Anti Vasemägi:** conceptualization (lead), data curation (equal), funding acquisition (lead), methodology (equal), project administration (equal), resources (lead), supervision (lead), validation (equal), visualization (equal), writing – original draft (lead), writing – review and editing (lead).

## Funding

This study was funded by the Swedish Research Council (grant 2020‐03916 to A.V.), the Estonian Research Council (grant PRG852 to R.G.), the Ella and Georg Ehrnrooth Foundation (to M.O.), and INTERACT (International Network for Terrestrial Research and Monitoring in the Arctic; to A.V.). Detailed information on funding sources supporting the collection and provision of samples by contributing partners is available in Table S5.

## Conflicts of Interest

The authors declare no conflicts of interest.

## Supporting information


**Figure S1:** (a) PCA plot for 269 *Perca fluviatilis* individuals with overlapping mtDNA and nDNA markers, highlighting evolutionary lineages. (b) Violin plot showing the distribution of pairwise *F*_ST_ estimates across 169 populations. (c) Boxplots illustrating heterozygosity observed (*H*_o_) from the SNP array for 363 individuals spanning 29 countries. BE, Belgium; BG, Bulgaria; CH, Switzerland; CZ, the Czech Republic; DE, Germany; DK, Denmark; EE, Estonia; ES, Spain; FI, Finland; FR, France; GB, England; GE, Georgia; HU, Hungary; IE, Ireland; LT, Lithuania; LV, Latvia; MK, North Macedonia; MN, Mongolia; NL, the Netherlands; PL, Poland; PT, Portugal; RO, Romania; RS, Serbia; RU, Russia; SE, Sweden; SI, Slovenia; SK, Slovakia; TR, Turkey; UA, Ukraine.

**Figure S2:** Ancestry inference of 363 *Perca fluviatilis* individuals based on nuclear SNPs, performed using ADMIXTURE with varying numbers of clusters (*K* = 2, 4, 6, 8, 10, 12).

**Figure S3:** (a, c) Isolation by distance (IBD) model plots constructed for mtDNA Lineages 3 and 4 alongside the corresponding Mantel correlograms (b, d).

**Figure S4:** Heatmap of mean pairwise *F*_ST_ values among countries, calculated from nuclear SNP array data. Values represent the average of all pairwise population‐level *F*_ST_ estimates between countries, based on populations with paired samples (*n* = 169 populations). BE, Belgium; BG, Bulgaria; CH, Switzerland; CZ, the Czech Republic; DE, Germany; DK, Denmark; EE, Estonia; ES, Spain; FI, Finland; FR, France; GB, England; GE, Georgia; HU, Hungary; IE, Ireland; LT, Lithuania; LV, Latvia; MK, North Macedonia; MN, Mongolia; NL, the Netherlands; PL, Poland; PT, Portugal; RO, Romania; RS, Serbia; RU, Russia; SE, Sweden; SI, Slovenia; SK, Slovakia; TR, Turkey; UA, Ukraine.

**Table S1:** Detailed information on 384 samples, including 269 samples that passed all tresholds for subsequent mtDNA and nuclear SNP analyses.

**Table S2:** Pairwise FST estimates between 169 populations with paired‐only individuals, nuclear SNP data*.

**Table S3:** Heterozygosity observed and nucleotide diversity estimates across 363 individuals.

**Table S4:** Pairwise comparisons of geographic and genetic distances (FST*) between 169 populations.

**Table S5:** List of sample providers, sampled countries, sampling methods, permit types, and grant information.

## Data Availability

All raw data used in this study during the review process are available on Zenodo (https://doi.org/10.5281/zenodo.17073118). The dataset includes a trimmed alignment of 269 mitochondrial DNA sequences in FASTA format and a SNP array comprising 363 individuals. In addition, all Supporting Information—S1, including five tables and four figures presented in, are deposited on Zenodo (https://doi.org/10.5281/zenodo.17127975).

## References

[ece373502-bib-0001] Albrechtsen, A. , F. C. Nielsen , and R. Nielsen . 2010. “Ascertainment Biases in SNP Chips Affect Measures of Population Divergence.” Molecular Biology and Evolution 27, no. 11: 2534–2547. 20558595 10.1093/molbev/msq148 PMC3107607

[ece373502-bib-0002] Alexander, D. H. , S. S. Shringarpure , J. Novembre , and K. Lange . 2015. Admixture 1.3 Software Manual. UCLA Human Genetics Software Distribution.

[ece373502-bib-0003] Allendorf, F. W. , P. A. Hohenlohe , and G. Luikart . 2010. “Genomics and the Future of Conservation Genetics.” Nature Reviews Genetics 11, no. 10: 697–709. 10.1038/nrg2844 20847747

[ece373502-bib-0004] Andersson, L. , D. Bekkevold , F. Berg , et al. 2024. “How Fish Population Genomics Can Promote Sustainable Fisheries: A Road Map.” Annual Review of Animal Biosciences 12, no. 1: 1–20. 37906837 10.1146/annurev-animal-021122-102933

[ece373502-bib-0005] Andrews, K. R. , J. M. Good , M. R. Miller , G. Luikart , and P. A. Hohenlohe . 2016. “Harnessing the Power of RADseq for Ecological and Evolutionary Genomics.” Nature Reviews Genetics 17, no. 2: 81–92. 10.1038/nrg.2015.28 PMC4823021 26729255

[ece373502-bib-0006] Andrews, S. F. 2010. A Quality Control Tool for High Throughput Sequence Data.

[ece373502-bib-0008] Avise, J. C. 2000. “The History and Purview of Phylogeography.” In Phylogeography: The History and Formation of Species, 3–25. Harvard University Press.

[ece373502-bib-0009] Bahr, A. , F. Lamy , H. Arz , H. Kuhlmann , and G. Wefer . 2005. “Late Glacial to Holocene Climate and Sedimentation History in the NW Black Sea.” Marine Geology 214, no. 4: 309–322.

[ece373502-bib-0010] Bennett, K. D. , P. C. Tzedakis , and K. J. Willis . 1991. “Quaternary Refugia of North European Trees.” Journal of Biogeography 18, no. 1: 103–115.

[ece373502-bib-0011] Bernatchez, L. , and C. C. Wilson . 1998. “Comparative Phylogeography of Nearctic and Palearctic Fishes.” Molecular Ecology 7, no. 4: 431–452.

[ece373502-bib-0012] Berthelsen, A. S. , M. Søndergaard , M. Kiljunen , and A. P. Eloranta . 2025. “Lauridsen, T. L. (2024). Pelagic Niche Shift by Fishes Following Restorations of a Eutrophic Lake.” Hydrobiologia 852: 289–303. 10.1007/s10750-024-05568-5.

[ece373502-bib-0013] Bhagwat, S. A. , and K. J. Willis . 2008. “Species Persistence in Northerly Glacial Refugia of Europe: A Matter of Chance or Biogeographical Traits?” Journal of Biogeography 35, no. 3: 464–482.

[ece373502-bib-0014] Bravo, G. A. , A. Antonelli , C. D. Bacon , et al. 2019. “Embracing Heterogeneity: Coalescing the Tree of Life and the Future of Phylogenomics.” PeerJ 7: e6399. 30783571 10.7717/peerj.6399 PMC6378093

[ece373502-bib-0015] Carlsson, J. , T. F. Cross , P. McGinnity , P. A. Prodöhl , and A. D. McDevitt . 2014. “The Use of Genetics to Infer the Origins of the Terrestrial and Freshwater Elements of the Irish Fauna.” Irish Naturalists' Journal 33: 8–27.

[ece373502-bib-0016] Chang, J. J. M. , M. J. Raupach , L. Cheng , et al. 2024. “Skimming the Skaters: Genome Skimming Improves Phylogenetic Resolution of *Halobatinae* (*Hemiptera: Gerridae*).” Insect Systematics and Diversity 8, no. 4: 3.

[ece373502-bib-0017] Chen, S. , Y. Zhou , Y. Chen , and J. Gu . 2018. “Fastp: An Ultra‐Fast All‐In‐One FASTQ Preprocessor.” Bioinformatics 34, no. 17: i884–i890. 30423086 10.1093/bioinformatics/bty560 PMC6129281

[ece373502-bib-0018] Christensen, E. A. , M. Grosell , and J. F. Steffensen . 2019. “Maximum Salinity Tolerance and Osmoregulatory Capabilities of European Perch *Perca fluviatilis* Populations Originating From Different Salinity Habitats. *Conservation* .” Physiology 7, no. 1: coz004. 10.1093/conphys/coz004 PMC6382033 30805189

[ece373502-bib-0019] Coll‐Costa, C. , C. Dahms , P. Kemppainen , et al. 2024. “Parallel Evolution Despite Low Genetic Diversity in Three‐Spined Sticklebacks.” Proceedings of the Royal Society B 291, no. 2020: 20232617. 38593844 10.1098/rspb.2023.2617 PMC11003780

[ece373502-bib-0020] Collette, B. B. , and P. Banarescu . 1977. “Systematics and Zoogeography of the Fishes of the Family *Percidae* .” Journal of the Fisheries Board of Canada 34, no. 10: 1450–1463.

[ece373502-bib-0021] Comes, H. P. , and J. W. Kadereit . 1998. “The Effect of Quaternary Climatic Changes on Plant Distribution and Evolution.” Trends in Plant Science 3, no. 11: 432–438.

[ece373502-bib-0022] Costedoat, C. , and A. Gilles . 2009. “Quaternary Pattern of Freshwater Fishes in Europe: Comparative Phylogeography and Conservation Perspective.” Open Conservation Biology Journal 3, no. 1: 36–48.

[ece373502-bib-0023] Crookes, S. , and P. W. Shaw . 2016. “Isolation by Distance and Non‐Identical Patterns of Gene Flow Within Two River Populations of the Freshwater Fish *Rutilus rutilus* (L. 1758).” Conservation Genetics 17, no. 4: 861–874. 32355467 10.1007/s10592-016-0828-3 PMC7175732

[ece373502-bib-0024] Devlin, S. P. , J. Saarenheimo , J. Syväranta , and R. I. Jones . 2015. “Top Consumer Abundance Influences Lake Methane Efflux.” Nature Communications 6, no. 1: 8787. 10.1038/ncomms9787 PMC4659926 26531291

[ece373502-bib-0025] Dias, D. , S. Batista , S. Nogueira , et al. 2025. “Integrating Social Media and Environmental DNA Records to Enhance Surveillance and Improve Early Detection of Invasive Species.” NeoBiota 102: 209–226.

[ece373502-bib-0026] Diehl, S. 1992. “Fish Predation and Benthic Community Structure: The Role of Omnivory and Habitat Complexity.” Ecology 73, no. 5: 1646–1661.

[ece373502-bib-0027] Dray, S. , and A.‐B. Dufour . 2007. “The ade4 Package: Implementing the Duality Diagram for Ecologists.” Journal of Statistical Software 22: 20.

[ece373502-bib-0028] Durand, J. D. , H. Persat , and Y. Bouvet . 1999. “Phylogeography and Postglacial Dispersion of the Chub ( *Leuciscus cephalus* ) in Europe.” Molecular Ecology 8, no. 6: 989–997. 10434419 10.1046/j.1365-294x.1999.00654.x

[ece373502-bib-0029] Edwards, S. V. , V. V. Robin , N. Ferrand , and C. Moritz . 2022. “The Evolution of Comparative Phylogeography: Putting the Geography (And More) Into Comparative Population Genomics.” Genome Biology and Evolution 14, no. 1: evab176. 34347070 10.1093/gbe/evab176 PMC8743039

[ece373502-bib-0030] Ekblom, R. , and J. Galindo . 2011. “Applications of Next Generation Sequencing in Molecular Ecology of Non‐Model Organisms.” Heredity 107, no. 1: 1–15. 21139633 10.1038/hdy.2010.152 PMC3186121

[ece373502-bib-0031] Ellegren, H. 2014. “Genome Sequencing and Population Genomics in Non‐Model Organisms.” Trends in Ecology & Evolution 29, no. 1: 51–63. 24139972 10.1016/j.tree.2013.09.008

[ece373502-bib-0032] Ellegren, H. , and N. Galtier . 2016. “Determinants of Genetic Diversity.” Nature Reviews Genetics 17, no. 7: 422–433. 10.1038/nrg.2016.58 27265362

[ece373502-bib-0033] Emerson, K. J. , C. R. Merz , J. M. Catchen , et al. 2010. “Resolving Postglacial Phylogeography Using High‐Throughput Sequencing.” Proceedings of the National Academy of Sciences 107, no. 37: 16196–16200. 10.1073/pnas.1006538107 PMC2941283 20798348

[ece373502-bib-0034] Eschbach, E. , A. W. Nolte , K. Kohlmann , J. Alós , S. Schöning , and R. Arlinghaus . 2021. “Genetic Population Structure of a Top Predatory Fish (Northern Pike, *Esox lucius* ) Covaries With Anthropogenic Alteration of Freshwater Ecosystems.” Freshwater Biology 66, no. 5: 884–901.

[ece373502-bib-0035] Eschbach, E. , A. W. Nolte , K. Kohlmann , P. Kersten , J. Kail , and R. Arlinghaus . 2014. “Population Differentiation of Zander ( *Sander lucioperca* ) Across Native and Newly Colonized Ranges Suggests Increasing Admixture in the Course of an Invasion.” Evolutionary Applications 7, no. 5: 555–568. 24944569 10.1111/eva.12155 PMC4055177

[ece373502-bib-0036] Falush, D. , M. Stephens , and J. K. Pritchard . 2003. “Inference of Population Structure Using Multilocus Genotype Data: Linked Loci and Correlated Allele Frequencies.” Genetics 164, no. 4: 1567–1587. 12930761 10.1093/genetics/164.4.1567 PMC1462648

[ece373502-bib-0037] Fuentes‐Pardo, A. P. , and D. E. Ruzzante . 2017. “Whole‐Genome Sequencing Approaches for Conservation Biology: Advantages, Limitations and Practical Recommendations.” Molecular Ecology 26, no. 20: 5369–5406. 28746784 10.1111/mec.14264

[ece373502-bib-0038] Fumagalli, M. 2013. “Assessing the Effect of Sequencing Depth and Sample Size in Population Genetics Inferences.” PLoS One 8, no. 11: e79667. 24260275 10.1371/journal.pone.0079667 PMC3832539

[ece373502-bib-0039] Gómez, A. , and D. H. Lunt . 2007. Refugia Within Refugia: Patterns of Phylogeographic Concordance in the Iberian Peninsula, 155–188. Evolutionary perspectives on the origins and conservation of European biodiversity.

[ece373502-bib-0040] Goodwin, S. , J. D. McPherson , and W. R. McCombie . 2016. “Coming of Age: Ten Years of Next‐Generation Sequencing Technologies.” Nature Reviews Genetics 17, no. 6: 333–351. 10.1038/nrg.2016.49 PMC10373632 27184599

[ece373502-bib-0041] Goslee, S. C. , and D. L. Urban . 2007. “The Ecodist Package for Dissimilarity‐Based Analysis of Ecological Data.” Journal of Statistical Software 22: 1–19.

[ece373502-bib-0043] Gum, B. , R. Gross , and R. Kuehn . 2005. “Mitochondrial and Nuclear DNA Phylogeography of European Grayling ( *Thymallus thymallus* ): Evidence for Secondary Contact Zones in Central Europe.” Molecular Ecology 14, no. 6: 1707–1725. 15836644 10.1111/j.1365-294X.2005.02520.x

[ece373502-bib-0044] Hewitt, G. 2000. “The Genetic Legacy of the Quaternary Ice Ages.” Nature 405, no. 6789: 907–913. 10879524 10.1038/35016000

[ece373502-bib-0045] Hewitt, G. M. 1996. “Some Genetic Consequences of Ice Ages, and Their Role in Divergence and Speciation.” Biological Journal of the Linnean Society 58, no. 3: 247–276.

[ece373502-bib-0046] Hewitt, G. M. 1999. “Post‐Glacial Re‐Colonization of European Biota.” Biological Journal of the Linnean Society 68, no. 1–2: 87–112.

[ece373502-bib-0047] Hewitt, G. M. 2004a. “Genetic Consequences of Climatic Oscillations in the Quaternary.” Philosophical Transactions of the Royal Society of London. Series B: Biological Sciences 359, no. 1442: 183–195. 15101575 10.1098/rstb.2003.1388 PMC1693318

[ece373502-bib-0117] Hewitt, G. M. 2004b. “The Structure of Biodiversity–Insights From Molecular Phylogeography.” Frontiers in Zoology 1, no. 1: 4. 15679920 10.1186/1742-9994-1-4 PMC544936

[ece373502-bib-0048] Hijmans, R. J. 2022. Geosphere: Spherical Trigonometry. R Package Version 1.5–18. https://CRAN.R‐project.org/package=geosphere.

[ece373502-bib-0049] Hughes, J. M. , D. J. Schmidt , and D. S. Finn . 2009. “Genes in Streams: Using DNA to Understand the Movement of Freshwater Fauna and Their Riverine Habitat.” Bioscience 59, no. 7: 573–583.

[ece373502-bib-0050] Jombart, T. 2008. “Adegenet: A R Package for the Multivariate Analysis of Genetic Markers.” Bioinformatics 24, no. 11: 1403–1405. 18397895 10.1093/bioinformatics/btn129

[ece373502-bib-0051] Jombart, T. , and I. Ahmed . 2011. “Adegenet 1.3‐1: New Tools for the Analysis of Genome‐Wide SNP Data.” Bioinformatics 27, no. 21: 3070–3071. 21926124 10.1093/bioinformatics/btr521 PMC3198581

[ece373502-bib-0052] Kaeuffer, R. , D. Réale , D. W. Coltman , and D. Pontier . 2007. “Detecting Population Structure Using STRUCTURE Software: Effect of Background Linkage Disequilibrium.” Heredity 99, no. 4: 374–380. 17622269 10.1038/sj.hdy.6801010

[ece373502-bib-0053] Kalyaanamoorthy, S. , B. Q. Minh , T. K. Wong , A. Von Haeseler , and L. S. Jermiin . 2017. “ModelFinder: Fast Model Selection for Accurate Phylogenetic Estimates.” Nature Methods 14, no. 6: 587–589. 10.1038/nmeth.4285. 28481363 PMC5453245

[ece373502-bib-0055] Kassambara, A. , and F. Mundt . 2020. Factoextra: Extract and Visualize the Results of Multivariate Data Analyses. R Package Version 1.0.7. https://CRAN.R‐project.org/package=factoextra.

[ece373502-bib-0056] Kontula, T. , and R. Väinölä . 2001. “Postglacial Colonization of Northern Europe by Distinct Phylogeographic Lineages of the Bullhead, *Cottus gobio* .” Molecular Ecology 10, no. 8: 1983. 11555242 10.1046/j.1365-294x.2001.01328.x

[ece373502-bib-0057] Kotlik, P. , and P. Berrebi . 2001. “Phylogeography of the Barbel ( *Barbus barbus* ) Assessed by Mitochondrial DNA Variation.” Molecular Ecology 10, no. 9: 2177–2185. 11555260 10.1046/j.0962-1083.2001.01344.x

[ece373502-bib-0058] Kuhl, H. , P. T. Euclide , C. Klopp , et al. 2024. “Multi‐Genome Comparisons Reveal Gain‐And‐Loss Evolution of Anti‐Mullerian Hormone Receptor Type 2 as a Candidate Master Sex‐Determining Gene in *Percidae* .” BMC Biology 22, no. 1: 141. 38926709 10.1186/s12915-024-01935-9 PMC11209984

[ece373502-bib-0059] Lachance, J. , and S. A. Tishkoff . 2013. “SNP Ascertainment Bias in Population Genetic Analyses: Why It Is Important, and How to Correct It.” BioEssays 35, no. 9: 780–786. 23836388 10.1002/bies.201300014 PMC3849385

[ece373502-bib-0060] Langmead, B. , and S. L. Salzberg . 2012. “Fast Gapped‐Read Alignment With Bowtie 2.” Nature Methods 9, no. 4: 357–359. 22388286 10.1038/nmeth.1923 PMC3322381

[ece373502-bib-0061] Letunic, I. , and P. Bork . 2019. “Interactive Tree of Life (iTOL) v4: Recent Updates and New Developments.” Nucleic Acids Research 47, no. W1: W256–W259. 30931475 10.1093/nar/gkz239 PMC6602468

[ece373502-bib-0062] Li, H. 2011. “A Statistical Framework for SNP Calling, Mutation Discovery, Association Mapping and Population Genetical Parameter Estimation From Sequencing Data.” Bioinformatics 27, no. 21: 2987–2993. 21903627 10.1093/bioinformatics/btr509 PMC3198575

[ece373502-bib-0063] Li, H. 2013. Seqtk: A Fast and Lightweight Tool for Processing FASTA or FASTQ Sequences. https://githubcom/lh3/seqtk.

[ece373502-bib-0064] Lichman, V. , M. Ozerov , M. E. López , et al. 2024. “Whole‐Genome Analysis Reveals Phylogenetic and Demographic History of Eurasian Perch.” Journal of Fish Biology 105, no. 3: 871–885. 38897597 10.1111/jfb.15821

[ece373502-bib-0065] López, M. E. , M. Ozerov , L. Pukk , K. Noreikiene , R. Gross , and A. Vasemägi . 2025. “Dynamic Outlier Slicing Allows Broader Exploration of Adaptive Divergence: A Comparison of Individual Genome and Pool‐Seq Data Linked to Humic Adaptation in Perch.” Molecular Ecology 34, no. 4: e17659. 39846218 10.1111/mec.17659 PMC11815547

[ece373502-bib-0067] Mangerud, J. , M. Jakobsson , H. Alexanderson , et al. 2004. “Ice‐Dammed Lakes and Rerouting of the Drainage of Northern Eurasia During the Last Glaciation.” Quaternary Science Reviews 23, no. 11–13: 1313–1332.

[ece373502-bib-0068] Médail, F. , and P. Quézel . 1999. “Biodiversity Hotspots in the Mediterranean Basin: Setting Global Conservation Priorities.” Conservation Biology 13, no. 6: 1510–1513.

[ece373502-bib-0069] Miles, A. , M. F. Rodrigues , P. Ralph , et al. 2024. “cggh/scikit‐allel: v1.3.8.” 10.5281/zenodo.10876220.

[ece373502-bib-0070] Minh, B. Q. , H. A. Schmidt , O. Chernomor , et al. 2020. “IQ‐TREE 2: New Models and Efficient Methods for Phylogenetic Inference in the Genomic Era.” Molecular Biology and Evolution 37, no. 5: 1530–1534. 10.1093/molbev/msaa015. 32011700 PMC7182206

[ece373502-bib-0071] Miraldo, A. , G. M. Hewitt , O. S. Paulo , and B. C. Emerson . 2011. “Phylogeography and Demographic History of *Lacerta lepida* in the Iberian Peninsula: Multiple Refugia, Range Expansions and Secondary Contact Zones.” BMC Evolutionary Biology 11: 1–19. 21682856 10.1186/1471-2148-11-170 PMC3141430

[ece373502-bib-0072] Myers, N. , R. A. Mittermeier , C. G. Mittermeier , G. A. B. da Fonseca , and J. Kent . 2000. “Biodiversity Hotspots for Conservation Priorities.” Nature 403: 853–858. 10706275 10.1038/35002501

[ece373502-bib-0073] Nazareno, A. G. , J. B. Bemmels , C. W. Dick , and L. G. Lohmann . 2017. “Minimum Sample Sizes for Population Genomics: An Empirical Study From an Amazonian Plant Species.” Molecular Ecology Resources 17, no. 6: 1136–1147. 28078808 10.1111/1755-0998.12654

[ece373502-bib-0074] Nesbø, C. L. , T. Fossheim , L. A. Vøllestad , and K. S. Jakobsen . 1999. “Genetic Divergence and Phylogeographic Relationships Among European Perch ( *Perca fluviatilis* ) Populations Reflect Glacial Refugia and Postglacial Colonization.” Molecular Ecology 8, no. 9: 1387–1404. 10564445 10.1046/j.1365-294x.1999.00699.x

[ece373502-bib-0075] Nilsson, J. , J. Andersson , P. Karås , and O. Sandström . 2004. “Recruitment Failure and Decreasing Catches of Perch ( *Perca fluviatilis* L.) and Pike ( *Esox lucius* L.) in the Coastal Waters of Southeast Sweden.” Boreal Environment Research 9, no. 4: 295.

[ece373502-bib-0076] Nordahl, O. , P. Koch‐Schmidt , J. Sunde , et al. 2019. “Genetic Differentiation Between and Within Ecotypes of Pike ( *Esox lucius* ) in the Baltic Sea.” Aquatic Conservation: Marine and Freshwater Ecosystems 29, no. 11: 1923–1935.

[ece373502-bib-0077] Noreikiene, K. , K. E. Aavik , S. Kahar , et al. 2024. “Eggs on a Plate: Population‐Dependent Effects of Humic Substance Rich Water on Perch Egg Size and Hatching Success.” Freshwater Biology 69, no. 9: 1307–1321.

[ece373502-bib-0078] Noreikiene, K. , M. Ozerov , F. Ahmad , et al. 2020. “Humic‐Acid‐Driven Escape From Eye Parasites Revealed by RNA‐Seq and Target‐Specific Metabarcoding.” Parasites & Vectors 13: 1–11. 32859251 10.1186/s13071-020-04306-9 PMC7456052

[ece373502-bib-0079] Oikonomou, A. , F. Leprieur , and I. D. Leonardos . 2014. “Biogeography of Freshwater Fishes of the Balkan Peninsula.” Hydrobiologia 738: 205–220.

[ece373502-bib-0080] Østbye, K. , P. A. Amundsen , L. Bernatchez , et al. 2006. “Parallel Evolution of Ecomorphological Traits in the European Whitefish *Coregonus lavaretus* (L.) Species Complex During Postglacial Times.” Molecular Ecology 15, no. 13: 3983–4001. 17054498 10.1111/j.1365-294X.2006.03062.x

[ece373502-bib-0081] Østbye, K. , L. Bernatchez , T. F. Næsje , K. J. Himberg , and K. Hindar . 2005. “Evolutionary History of the European Whitefish *Coregonus lavaretus* (L.) Species Complex as Inferred From mtDNA Phylogeography and Gill‐Raker Numbers.” Molecular Ecology 14, no. 14: 4371–4387. 16313599 10.1111/j.1365-294X.2005.02737.x

[ece373502-bib-0082] Ouborg, N. J. , C. Pertoldi , V. Loeschcke , R. K. Bijlsma , and P. W. Hedrick . 2010. “Conservation Genetics in Transition to Conservation Genomics.” Trends in Genetics 26, no. 4: 177–187. 20227782 10.1016/j.tig.2010.01.001

[ece373502-bib-0083] Ozerov, M. , K. Noreikiene , S. Kahar , et al. 2022. “Whole‐Genome Sequencing Illuminates Multifaceted Targets of Selection to Humic Substances in Eurasian Perch.” Molecular Ecology 31, no. 8: 2367–2383. 35202502 10.1111/mec.16409 PMC9314028

[ece373502-bib-0085] Ozerov, M. Y. , F. Ahmad , R. Gross , et al. 2018. “Highly Continuous Genome Assembly of Eurasian Perch ( *Perca fluviatilis* ) Using Linked‐Read Sequencing.” G3: Genes, Genomes, Genetics 8, no. 12: 3737–3743. 30355765 10.1534/g3.118.200768 PMC6288837

[ece373502-bib-0303] Ozerov, M. Y. , K. Noreikiene , K. Taube , R. Gross , and A. Vasemägi . 2025. “Integrative Genomics Refines Tissues, Candidate Genes and Putative Regulatory Links Involved in the Humic Adaptation of Keystone Freshwater Fish.” Molecular Ecology 34, no. 15: e17698. 39964025 10.1111/mec.17698 PMC12288802

[ece373502-bib-0086] Purcell, S. , B. Neale , K. Todd‐Brown , et al. 2007. “PLINK: A Tool Set for Whole‐Genome Association and Population‐Based Linkage Analyses.” American Journal of Human Genetics 81, no. 3: 559–575. 17701901 10.1086/519795 PMC1950838

[ece373502-bib-0118] R Core Team . 2022. “R: A Language and Environment for Statistical Computing (Version 4.1.3).” R Foundation for Statistical Computing. https://www.R‐project.org/.

[ece373502-bib-0087] Reyjol, Y. , B. Hugueny , D. Pont , et al. 2007. “Patterns in Species Richness and Endemism of European Freshwater Fish.” Global Ecology and Biogeography 16, no. 1: 65–75.

[ece373502-bib-0088] Ribeiro, F. , M. J. Collares‐Pereira , and P. B. Moyle . 2009. “Non‐Native Fish in the Fresh Waters of Portugal, Azores and Madeira Islands: A Growing Threat to Aquatic Biodiversity.” Fisheries Management and Ecology 16, no. 4: 255–264.

[ece373502-bib-0089] Roques, C. , M. Zahm , C. Klopp , O. Bouche , C. Donnadieu , and H. Kuhl . 2020. *Perca fluviatilis* Genome Sequencing and Assembly. NCBI. https://www.ncbi.nlm.nih.gov/datasets/genome/GCF_010015445.1/.

[ece373502-bib-0090] Rousset, F. 1997. “Genetic Differentiation and Estimation of Gene Flow From F‐Statistics Under Isolation by Distance.” Genetics 145, no. 4: 1219–1228. 9093870 10.1093/genetics/145.4.1219 PMC1207888

[ece373502-bib-0091] Salvi, D. , D. J. Harris , A. Kaliontzopoulou , M. A. Carretero , and C. Pinho . 2013. “Persistence Across Pleistocene Ice Ages in Mediterranean and Extra‐Mediterranean Refugia: Phylogeographic Insights From the Common Wall Lizard.” BMC Evolutionary Biology 13: 1–18. 23841475 10.1186/1471-2148-13-147 PMC3711914

[ece373502-bib-0092] Schmidt, B. V. , and J. Schaefer . 2018. “Ecological and Landscape Effects on Genetic Distance in an Assemblage of Headwater Fishes.” Ecology of Freshwater Fish 27, no. 2: 617–631.

[ece373502-bib-0093] Schmitt, T. 2007. “Molecular Biogeography of Europe: Pleistocene Cycles and Postglacial Trends.” Frontiers in Zoology 4: 11. 17439649 10.1186/1742-9994-4-11 PMC1868914

[ece373502-bib-0094] Schmitt, T. , and Z. Varga . 2012. “Extra‐Mediterranean Refugia: The Rule and Not the Exception?” Frontiers in Zoology 9, no. 1: 22. 22953783 10.1186/1742-9994-9-22 PMC3462695

[ece373502-bib-0095] Schönswetter, P. , I. Stehlik , R. Holderegger , and A. Tribsch . 2005. “Molecular Evidence for Glacial Refugia of Mountain Plants in the European Alps.” Molecular Ecology 14, no. 11: 3547–3555. 16156822 10.1111/j.1365-294X.2005.02683.x

[ece373502-bib-0096] Shafer, A. B. , J. B. Wolf , P. C. Alves , et al. 2015. “Genomics and the Challenging Translation Into Conservation Practice.” Trends in Ecology & Evolution 30, no. 2: 78–87. 25534246 10.1016/j.tree.2014.11.009

[ece373502-bib-0097] Skovrind, M. , G. Pacheco , E. A. F. Christensen , et al. 2023. “Uncovering Neutral and Adaptive Genomic Differentiation Among European Perch With Brackish Water and Freshwater Origin in the Western Baltic Sea Region.” Fisheries Research 268: 106846.

[ece373502-bib-0098] Sommer, R. S. , and A. Nadachowski . 2006. “Glacial Refugia of Mammals in Europe: Evidence From Fossil Records.” Mammal Review 36, no. 4: 251–265.

[ece373502-bib-0099] Stepien, C. A. , and A. E. Haponski . 2015. “Taxonomy, Distribution, and Evolution of the *Percidae* .” In Biology and Culture of Percid Fishes: Principles and Practices, 3–60. Springer Netherlands.

[ece373502-bib-0100] Stewart, J. R. , and A. M. Lister . 2001. “Cryptic Northern Refugia and the Origins of the Modern Biota.” Trends in Ecology & Evolution 16, no. 11: 608–613.

[ece373502-bib-0101] Stewart, J. R. , A. M. Lister , I. Barnes , and L. Dalén . 2010. “Refugia Revisited: Individualistic Responses of Species in Space and Time.” Proceedings of the Royal Society B: Biological Sciences 277, no. 1682: 661–671. 10.1098/rspb.2009.1272 PMC2842738 19864280

[ece373502-bib-0102] Stroeven, A. P. , C. Hättestrand , J. Kleman , et al. 2016. “Deglaciation of Fennoscandia.” Quaternary Science Reviews 147: 91–121.

[ece373502-bib-0103] Sümegi, P. , S. Gulyás , D. Molnár , et al. 2018. Ice Age Terrestrial and Freshwater Gastropod Refugia in the Carpathian Basin, Central Europe, 93–117. *Biological Resources of Water* .

[ece373502-bib-0104] Swatdipong, A. , A. Vasemägi , M. T. Koskinen , J. Piironen , and C. R. Primmer . 2009. “Unanticipated Population Structure of European Grayling in Its Northern Distribution: Implications for Conservation Prioritization.” Frontiers in Zoology 6: 1–12. 19331654 10.1186/1742-9994-6-6 PMC2676281

[ece373502-bib-0105] Taberlet, P. , L. Fumagalli , A. G. Wust‐Saucy , and J. F. Cosson . 1998. “Comparative Phylogeography and Postglacial Colonization Routes in Europe.” Molecular Ecology 7, no. 4: 453–464. 9628000 10.1046/j.1365-294x.1998.00289.x

[ece373502-bib-0106] Taube, K. , K. Noreikiene , S. Kahar , R. Gross , M. Ozerov , and A. Vasemägi . 2023. “Subtle Transcriptomic Response of Eurasian Perch ( *Perca fluviatilis* ) Associated With *Triaenophorus nodulosus* Plerocercoid Infection.” International Journal for Parasitology: Parasites and Wildlife 22: 146–154. 37869060 10.1016/j.ijppaw.2023.09.009 PMC10585624

[ece373502-bib-0107] Testo, W. 2021. Visualizing Speciation Rates With IDW Interpolation. https://github.com/wtesto/Speciation‐Rate‐Interpolation.

[ece373502-bib-0108] Thorpe, J. E. 1977. “Morphology, Physiology, Behavior, and Ecology of *Perca fluviatilis* L. and *P. flavescens* Mitchill.” Journal of the Fisheries Board of Canada 34, no. 10: 1504–1514.

[ece373502-bib-0109] Toomey, L. , S. Dellicour , T. Vanina , et al. 2020. “Getting Off on the Right Foot: Integration of Spatial Distribution of Genetic Variability for Aquaculture Development and Regulations, the European Perch Case.” Aquaculture 521: 734981.

[ece373502-bib-0110] Vasemägi, A. , M. Ozerov , K. Noreikiene , M. E. López , and A. Gårdmark . 2023. “Unlocking the Genome of Perch–From Genes to Ecology and Back Again.” Ecology of Freshwater Fish 32, no. 4: 677–702.

[ece373502-bib-0111] Vejřík, L. , I. Matějíčková , T. Jůza , et al. 2016. “Small Fish Use the Hypoxic Pelagic Zone as a Refuge From Predators.” Freshwater Biology 61, no. 6: 899–913.

[ece373502-bib-0112] Vörös, J. , P. Mikulíček , Á. Major , E. Recuero , and J. W. Arntzen . 2016. “Phylogeographic Analysis Reveals Northerly Refugia for the Riverine Amphibian *Triturus dobrogicus* (*Caudata: Salamandridae*).” Biological Journal of the Linnean Society 119, no. 4: 974–991.

[ece373502-bib-0113] Washburn, B. A. , M. F. Cashner , and R. E. Blanton . 2020. “Small Fish, Large River: Surprisingly Minimal Genetic Structure in a Dispersal‐Limited, Habitat Specialist Fish.” Ecology and Evolution 10, no. 4: 2253–2268. 32128153 10.1002/ece3.6064 PMC7042738

[ece373502-bib-0114] Weiss, S. , K. Schwenk , and C. Sturmbauer . 2018. “Phylogeography and Postglacial Colonization of European Freshwater Fishes.” Ecology and Evolution 8, no. 15: 7536–7548.

[ece373502-bib-0115] Willing, E. M. , C. Dreyer , and C. van Oosterhout . 2012. “Estimates of Genetic Differentiation Measured by *F* _ST_ Do Not Necessarily Require Large Sample Sizes When Using Many SNP Markers.” PLoS One 7, no. 8: e42649. 22905157 10.1371/journal.pone.0042649 PMC3419229

[ece373502-bib-0116] Wofford, J. E. , R. E. Gresswell , and M. A. Banks . 2005. “Influence of Barriers to Movement on Within‐Watershed Genetic Variation of Coastal Cutthroat Trout.” Ecological Applications 15, no. 2: 628–637.

