# Supplementary figures and images for: Pan‐Continental Genomic Analysis of Eurasian Perch Uncovers Global Diversity Hotspots and Postglacial Recolonization Patterns
Source: Ecol Evol. 2026 Apr 21;16(4):e73502. doi: 10.1002/ece3.73502 (PMC13099172; doi:10.1002/ece3.73502)

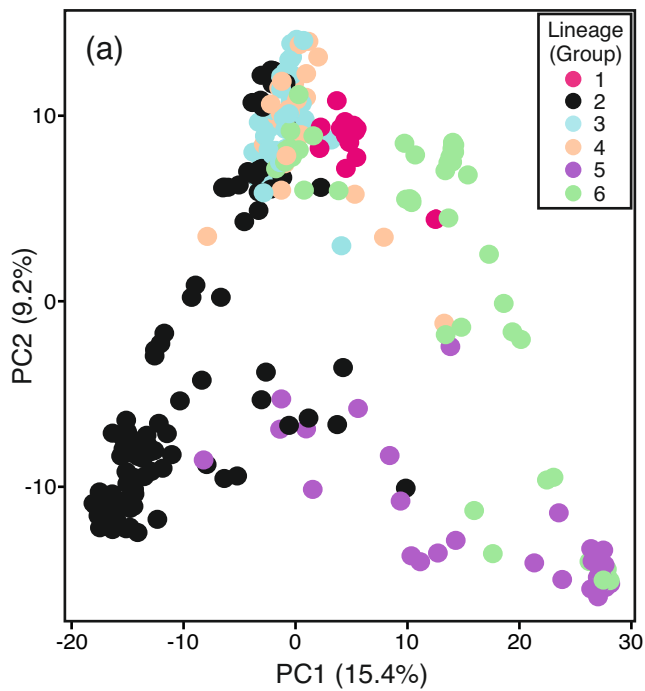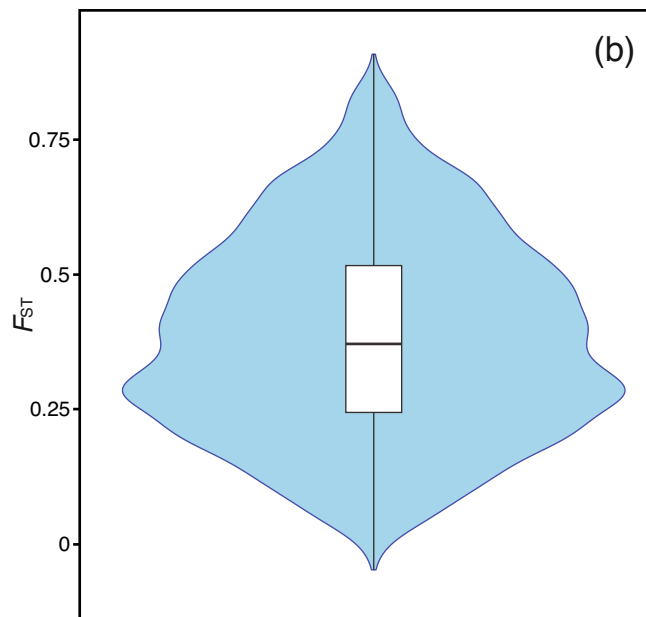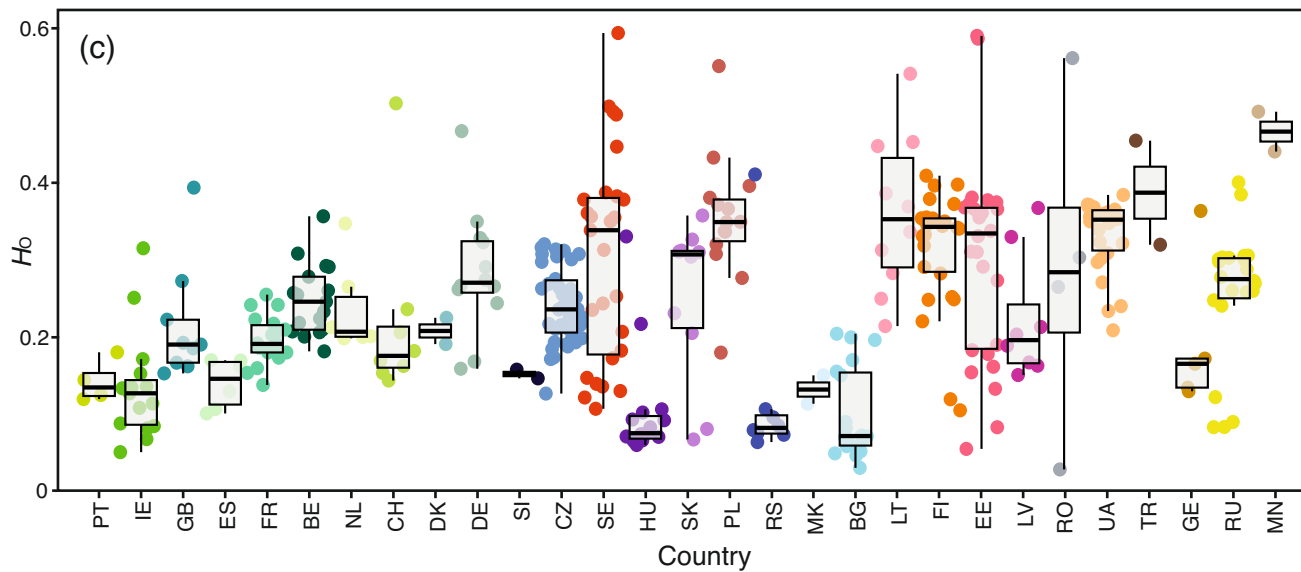

Supplement: Supplementary file 1 — Figure S1: (a) PCA plot for 269 Perca fluviatilis individuals with overlapping mtDNA and nDNA markers, highlighting evolutionary lineages. (b) Violin plot showing the distribution of pairwise F ST estimates across 169 populations. (c) Boxplots illustrating heterozygosity observed (H o) from the SNP array for 363 individuals spanning 29 countries. BE, Belgium; BG, Bulgaria; CH, Switzerland; CZ, the Czech Republic; DE, Germany; DK, Denmark; EE, Estonia; ES, Spain; FI, Finland; FR, France; GB, England; GE, Georgia; HU, Hungary; IE, Ireland; LT, Lithuania; LV, Latvia; MK, North Macedonia; MN, Mongolia; NL, the Netherlands; PL, Poland; PT, Portugal; RO, Romania; RS, Serbia; RU, Russia; SE, Sweden; SI, Slovenia; SK, Slovakia; TR, Turkey; UA, Ukraine. [file ECE3-16-e73502-s006.pdf]

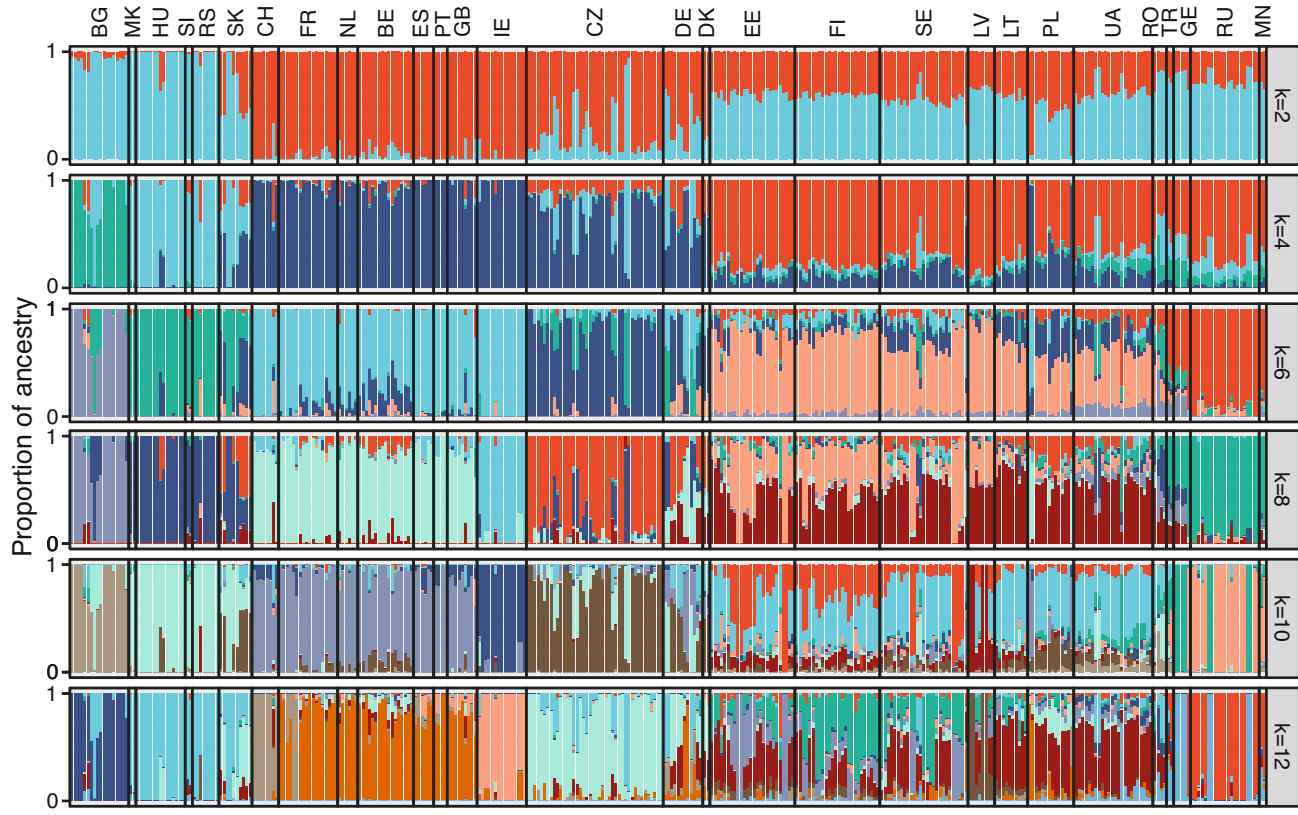

Supplement: Supplementary file 2 — Figure S2: Ancestry inference of 363 Perca fluviatilis individuals based on nuclear SNPs, performed using ADMIXTURE with varying numbers of clusters (K = 2, 4, 6, 8, 10, 12). [file ECE3-16-e73502-s007.pdf]

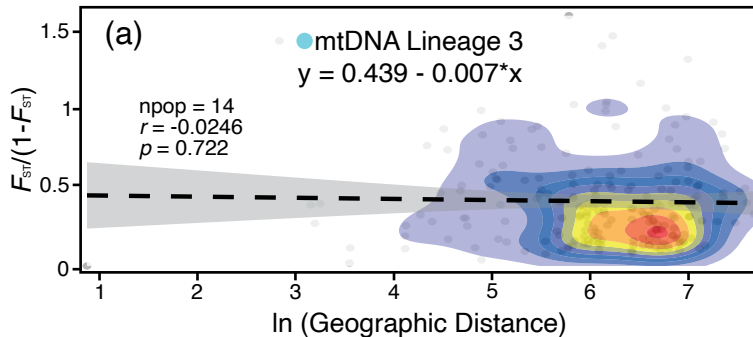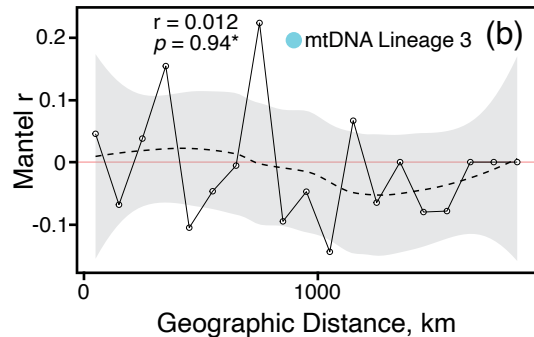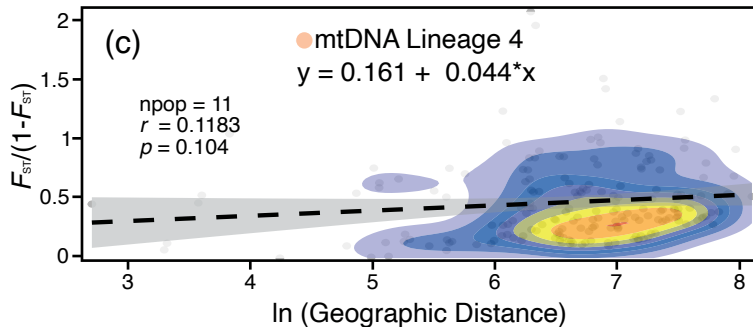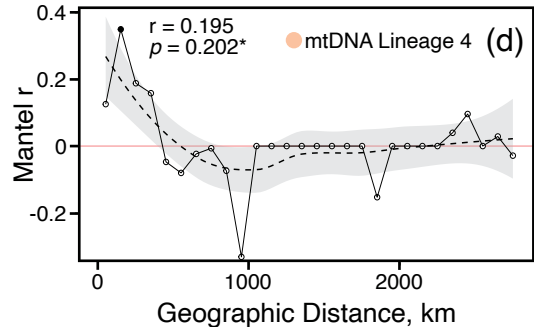

Supplement: Supplementary file 3 — Figure S3: (a, c) Isolation by distance (IBD) model plots constructed for mtDNA Lineages 3 and 4 alongside the corresponding Mantel correlograms (b, d). [file ECE3-16-e73502-s009.pdf]

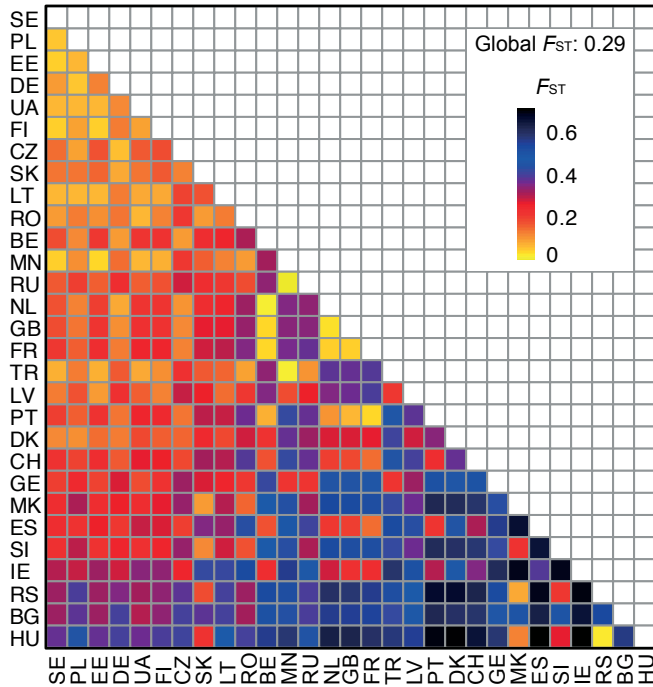

Supplement: Supplementary file 4 — Figure S4: Heatmap of mean pairwise F ST values among countries, calculated from nuclear SNP array data. Values represent the average of all pairwise population‐level F ST estimates between countries, based on populations with paired samples (n = 169 populations). BE, Belgium; BG, Bulgaria; CH, Switzerland; CZ, the Czech Republic; DE, Germany; DK, Denmark; EE, Estonia; ES, Spain; FI, Finland; FR, France; GB, England; GE, Georgia; HU, Hungary; IE, Ireland; LT, Lithuania; LV, Latvia; MK, North Macedonia; MN, Mongolia; NL, the Netherlands; PL, Poland; PT, Portugal; RO, Romania; RS, Serbia; RU, Russia; SE, Sweden; SI, Slovenia; SK, Slovakia; TR, Turkey; UA, Ukraine. [file ECE3-16-e73502-s003.pdf]
