# Supplementary material for: Pan‐Continental Genomic Analysis of Eurasian Perch Uncovers Global Diversity Hotspots and Postglacial Recolonization Patterns
Source: Ecol Evol. 2026 Apr 21;16(4):e73502. doi: 10.1002/ece3.73502 (PMC13099172; doi:10.1002/ece3.73502)
Supplement: Supplementary file 9 — Table S5: List of sample providers, sampled countries, sampling methods, permit types, and grant information. [file ECE3-16-e73502-s002.docx]

**Table S5**. List of sample providers, sampled countries, sampling methods, permit types, and grant information.

| **Sample Provider** | **Countries** | **Sampling Method** | **Permit** | **Funding** |
| --- | --- | --- | --- | --- |
| Aleš Snoj | Slovenia | Fishing rod | Fishing license | Not available |
| Anti Vasemägi | Estonia, Finland, Sweden | Gill- net, beach seine, recreational fishing gear | Sweden: Dnr 5.8.18-03449/2017; Estonian Ministry of Environment (54/2016) | Estonian Research Council grant PRG852 (to R.G.), Swedish Research Council grant 2020-03916 (to A.V.), the Ella and Georg Ehrnrooth foundation (to M.O.), INTERACT (International Network for Terrestrial Research and Monitoring in the Arctic, to A. V.) |
| Bella Japoshvili | Georgia | Recreational fishing gear, Gill nets | National fishing regulations,  permits N2501/01, N5768/01 | [Ministry of Education, Science and Youth of Georgia,](https://mes.gov.ge/?lang=eng)  Institute of Zoology |
| Dijana Blazhekovikj-Dimovska | North Macedonia | Recreational fishing gear | Provided by local fishermen | Not available |
| Dmitry Lajus | Russia | Recreational fishing gear | National fishing regulations and ethical guidelines for recreational fishing | Not available |
| Dušan Nikolić | Serbia | Standing gillnets (30 m × 2 m, 30-40 mm mesh size) | Ministry of Environmental Protection of the Republic of Serbia (No. 324-04-0224/2021-04) and Environment Protection Institute of Serbia (No. 026-74/2) | Ministry of Science, Technological Development and Innovation of the Republic of Serbia (Grant number: 451-03-33/2026-03/200053) |
| Filipe Ribeiro | Portugal (mainland)  Spain  Czech Republic  Romania | Gill netting with 20mm, 25mm and 30 mm mesh size (PT); Electric fishing (PT, ES, CZ), Fish Market (RO) | Institute for Conservation of Nature and Forestry (Nr. 218 and 219/2017/CAPT) | Ministry of Science and Technology, funded through Foundation for Science and Technology, FRISK project (Ref. PTDC/AAG-MAA/0350/2014) |
| Pedro Raposeiro | Portugal (Azores) | Gill netting | Secretaria Regional do Mar, Ciência e Tecnologia  CCPI25/2018/DRCT | Not available |
| Hugo Verreycken | Belgium | Double fyke nets, electrofishing | Permit from the Agency for Nature and Forestry d.d. 18 January 2021 | Not available |
| Robert Britton | England | Electric fishing, seine netting | Environment Agency (England) consents (Section 27a consent) | Not available |
| Jost Borcheling | Germany | Beach seining, recreational fishing gear | National fishing regulations and ethical guidelines for recreational fishing, permission by the Rheinfischerei­genossenschaft NRW | Not available |
| Yuliia Kutsokon / Oleksandr Didenko | Ukraine | Recreational fishing gear | National regulations guidelines for recreational fishing | Not available |
| Pavel Jurajda | Czech Republic | Gill netting, electrofishing | Electrofishing license 68/2023 | Not available |
| Rafael Miranda | Spain | Standard electrofishing | Forestry Service, Department of Agriculture, Foral Deputation of Álava (Basque Country, Spain) | Not available |
| Jan Kouřil | Mongolia, Poland, Russia, Slovakia, Ukraine | Recreational fishing gear | National fishing regulations and ethical guidelines for recreational fishing and fish handling as recommended by the respective countries' fishing federations | Ministry of Education, Youth and Sports of the Czech Republic – project “CENAKVA” (LM2018099) |
| Jens Carlsson | Ireland | European CEN standard gill netting. Multi-mesh monofilament survey gill nets (12 panel, 5-55mm mesh size) using a stratified random sampling design (CEN, 2015) were deployed | Dept. of Environment, climate and communications, certified under Section 14 of the Fisheries (Consolidation) ACT, 1959 as substituted by Section 4 of the Fisheries amendment ACT 1962. | Funded through Irish exchequer |
| Jiří Peterka | Czech Republic, Netherlands | European CEN standard gill netting | Evides water company N.V. | Not available |
| Kimmo Kahilainen | Finland | Recreational fishing, gill nets | No ethical permission required according to the Finnish Animal Conservation Law (7§ 28.6.2013/498) | Ministry of Agriculture and Forestry, European Regional Development Fund (project #A30205) and the Academy of Finland (grants #140903, #1268566) |
| L. Ložys/  K. Noreikiene | Lithuania | Recreational fishing gear | National fishing regulations and ethical guidelines for recreational fishing | Not available |
| Lilian Pukk | Estonia | Recreational fishing gear | Estonian Ministry of Environment (54/2016) | Estonian Research Council grant PRG852 (to R.G.) |
| Özgen Yilmaz | Turkey | Recreational fishing gear | Purchased from local fishermen before being preserved in 99% ethanol | Not available |
| Petr Blabolil | Czech Republic | Recreational fishing, electrofishing fyke nets | Czech anglers union permit No 00147677, electrofishing license No 14-3/17A | National Agency of Agricultural Research, (QL25020052 HOBIHO) |
| Peter Eklöv | Sweden | NORDIC nets | C231/10 | Swedish Research Council, Dnr. 621-2009-4764 |
| Péter Takács | Hungary | Standard electrofishing | Ministry of Agriculture, Department of Fisheries (permit № HAGF/79/2022) | National Multidisciplinary Laboratory for Climate Change (RRF-2.3.1-21-2022-00014) project within the framework of Hungary's National Recovery and Resilience Plan |
| Siim Kahar | Estonia, Latvia | Recreational fishing gear, gill- net | Estonian Ministry of Environment (54/2016), national fishing regulations | Estonian Research Council grant PRG852 (to R.G.) |
| Stefan Kazakov | Bulgaria | Recreational fishing gear (rod and line); electrofishing according to standard CEN EN 14011:2003 | National permit № 15/17.05.2025 (Ministry of Agriculture and Food of Bulgaria) | Not available |
| T. L. Lauridsen / A. Berthelsen | Denmark | Nordic multi-mesh gillnets (Berthelsen *et al*., 2024) | In compliance with Danish animal welfare laws (Berthelsen *et al*., 2024) | European Union’s Horizon 2020 research and innovation program under grant agreement No 951963 |
| Tatyana Gebauer | Lithuania, Poland, Slovakia | Recreational fishing gear | National fishing regulations and ethical guidelines for recreational fishing and fish handling as recommended by the respective countries' fishing federations | Ministry of Education, Youth and Sports of the Czech Republic – project “CENAKVA” (LM2018099); VVI CENAKVA Research Infrastructure (ID 90238, MEYS CR, 2023–2026) |
| Thomas Lecocq | Belgium, Finland, France,  Germany, Hungary, Poland, Romania, Switzerland, Russia,  England, Ukraine | Obtained through activities carried out for other purposes by local partners or purchased from local fishermen before being preserved in 99% ethanol | All samples were collected in compliance with both European and national sampling regulations; collection did not occur in privately-owned areas, protected locations, or involve protected species | Grand Est, France (DomPop project), and the French Ministère de l'Enseignement Supérieur et de la Recherche |
